# Sub-chronic inhalation of lead oxide nanoparticles revealed their broad distribution and tissue-specific subcellular localization in target organs

**DOI:** 10.1186/s12989-017-0236-y

**Published:** 2017-12-21

**Authors:** J. Dumková, T. Smutná, L. Vrlíková, P. Le Coustumer, Z. Večeřa, B. Dočekal, P. Mikuška, L. Čapka, P. Fictum, A. Hampl, M. Buchtová

**Affiliations:** 1 Department of Histology and Embryology, Faculty of Medicine, Masaryk University, 625 00 Brno, Czech Republic; 2 Institute of Animal Physiology and Genetics of the Czech Academy of Sciences, 602 00 Brno, Czech Republic; 3 Bordeaux University, UF STE, Allée G. Saint-Hilaire, 33615 Pessac Cedex, France; 4 UMR 5254 IPREM, CNRS/UPPA, Technopole Hélioparc, 2 av P. Angot, 64053 Pau Cedex9, France; 5 EA 4592 Georessources & Environnement/ Bordeaux Montaigne University-IPNB ENSEGID, Allée F. Daguin, 33615 Pessac Cedex, France; 6 Institute of Analytical Chemistry of the Czech Academy of Sciences, 602 00 Brno, Czech Republic; 7 Department of Pathological Morphology and Parasitology, Faculty of Veterinary Medicine, University of Veterinary and Pharmaceutical Sciences, 612 42 Brno, Czech Republic; 8 Department of Animal Physiology and Immunology, Institute of Experimental Biology, Faculty of Science, Masaryk University, 625 00 Brno, Czech Republic

**Keywords:** Nanoparticles, Lead oxide, Electron microscopy, Toxicity, Inhalation, Lung, Liver, Kidney, Spleen, Brain

## Abstract

**Background:**

Lead is well known environmental pollutant, which can cause toxic effects in multiple organ systems. However, the influence of lead oxide nanoparticles, frequently emitted to the environment by high temperature technological processes, is still concealed. Therefore, we investigate lead oxide nanoparticle distribution through the body upon their entry into lungs and determine the microscopic and ultramicroscopic changes caused by the nanoparticles in primary and secondary target organs.

**Methods:**

Adult female mice (ICR strain) were continuously exposed to lead oxide nanoparticles (PbO-NPs) with an average concentration approximately 10^6^ particles/cm^3^ for 6 weeks (24 h/day, 7 days/week). At the end of the exposure period, lung, brain, liver, kidney, spleen, and blood were collected for chemical, histological, immunohistochemical and electron microscopic analyses.

**Results:**

Lead content was found to be the highest in the kidney and lungs, followed by the liver and spleen; the smallest content of lead was found in brain. Nanoparticles were located in all analysed tissues and their highest number was found in the lung and liver. Kidney, spleen and brain contained lower number of nanoparticles, being about the same in all three organs. Lungs of animals exposed to lead oxide nanoparticles exhibited hyperaemia, small areas of atelectasis, alveolar emphysema, focal acute catarrhal bronchiolitis and also haemostasis with presence of siderophages in some animals. Nanoparticles were located in phagosomes or formed clusters within cytoplasmic vesicles. In the liver, lead oxide nanoparticle exposure caused hepatic remodeling with enlargement and hydropic degeneration of hepatocytes, centrilobular hypertrophy of hepatocytes with karyomegaly, areas of hepatic necrosis, occasional periportal inflammation, and extensive accumulation of lipid droplets. Nanoparticles were accumulated within mitochondria and peroxisomes forming aggregates enveloped by an electron-dense mitochondrial matrix. Only in some kidney samples, we observed areas of inflammatory infiltrates around renal corpuscles, tubules or vessels in the cortex. Lead oxide nanoparticles were dispersed in the cytoplasm, but not within cell organelles. There were no significant morphological changes in the spleen as a secondary target organ. Thus, pathological changes correlated with the amount of nanoparticles found in cells rather than with the concentration of lead in a given organ.

**Conclusions:**

Sub-chronic exposure to lead oxide nanoparticles has profound negative effects at both cellular and tissue levels. Notably, the fate and arrangement of lead oxide nanoparticles were dependent on the type of organs.

**Electronic supplementary material:**

The online version of this article (10.1186/s12989-017-0236-y) contains supplementary material, which is available to authorized users.

## Background

Lead is an environmental pollutant with the largest toxicological database [1] as it has been a known toxicant for thousands of years. Moreover, lead continues to remain a persistent environmental health threat today. Exposure to lead can result in significant adverse health effects in multiple organ systems. It’s toxic effects on the nervous, hematological, renal and reproductive systems have been studied extensively and have been documented in detail [2].

Lead originates from various industrial and/or household sources, and enters the body through food and fluid intakes, as well as by inhalation [3–5]. Children have been shown to be at greatest risk because of their enhanced gastrointestinal absorption of lead (40–50% vs. 10–15% in adults) [6] and an incompletely developed blood-brain barrier [3]. Lead exposure increases the risk of diminished intelligence, attention deficit, hyperactivity disorder, school failure and criminal behaviour, thus, there is no known safe level of exposure to lead [1].

Acute lead poisoning is characterized by non-specific symptoms such as abdominal pain (lead colic), joint pain, constipation, anorexia, muscle aches, headaches, decreased libido, sleep disturbance, irritability, fatigue, anemia, nephropathy, confusion, encephalopathy and seizures [7]. Chronic lead poisoning is the consequence of long-term exposure and can be asymptomatic or may be manifested by a spectrum of cardiovascular and neuropsychiatric diseases, and associated with increased risk of death.

Absorbed lead, which is not excreted out of body, is deposited primarily in mineralizing tissues (bones and teeth), which typically store the majority of the body burden of lead, and in soft tissue organs such as the liver, kidneys, lungs, brain, spleen, muscles and heart [8]. At a steady state, about 90% of lead in the human body was found in the skeleton of adults [2] and 73% in children, respectively [4]. The half-life of lead in adult human blood has been estimated to range between 28 and 36 days [8]. Lead is excreted via the kidneys with a half-life of about 30 days under normal kidney function. Skeletal lead has a half-life of decades [9], however, may be gradually released into the bloodstream even after exposure has ceased, particularly during physiological or pathological bone demineralization (such as pregnancy or lactation) [4].

While the overall negative effect of lead is well known, here we focused our investigation on influence of inhalation of lead oxide nanoparticles (PbO-NPs) on several selected organs. We selected nanoparticles with a diameter of less than 100 nm. Such nanoparticles can represent a serious issue for human health, which was previously shown for similarly sized nanoparticles of different chemistry, because of their easy transport through the cellular barriers [10–12]. Moreover, lead oxide nanoparticles are attention-grabbing substantial for further study as this toxic material is frequently emitted to the environment by high temperature technological processes, such as those used in sintering plants [13], lead smelters [5] and electric steel plants [14], where the lead oxide nanoparticles arise as a result of oxidation process of lead vapors due to the presence of oxygen.

As the respiratory track is one of the most common routes of human exposure to NPs [15], inhalation of lead oxide nanoparticles was selected for detailed examination of the effect on primary and secondary organs, which have been linked to accumulation, processing or metabolizing the toxins. In this study, we exposed female mice to PbO-NPs by inhalation in a whole body inhalation chamber representing physiological form of exposure similar to real conditions, which is in contrast to intratracheal instillation. In two independent experiments, we used approximately the same average mass concentration of lead oxide nanoparticles (121.7 μg PbO/m^3^; resp. 149.3 μg PbO/m^3^). Occupational Safety and Health Administration’s (OSHA) set a Permissible Exposure Limit (PEL) for lead in workplace air of 50 μg/m^3^ (8-h time weighted average) [16]. However, in urban areas and some megacities in India or China [17, 18], concentrations of coarse particulate matter PM_10_ (PM ≤ 10 μm; containing also metals from anthropogenic sources as Pb, Cd, Zn, Bi, Sb, Cu) can reach pollution peaks at >250 μg/m^3^ [19]. Thus, mass concentration of nanoparticles applied to our experimental animals was approximately on the same level, which is inhaled by inhabitants of megacities during pollution peaks and also corresponding to the exposure of lead workers in industrial areas.

The aim of this in vivo study is to follow the fate of lead oxide nanoparticles upon entry via the lungs, with the main focus on uncovering their localization and microscopic/ultramicroscopic changes caused by these nanoparticles to target soft tissues.

## Methods

### Animals

Adult female mice (ICR strain) were obtained from the Animal Facility of the Masaryk University (Brno, Czech Republic) and were allowed to acclimatize to laboratory conditions for at least one week before the inhalation experiments. Commercial feed and water were provided ad libitum.

### Preparation of PbO nanoparticles

PbO nanoparticles (PbO-NPs) were generated continuously in situ in a hot-wall tube flow reactor using an evaporation–oxidation–condensation technique in which a ceramic crucible containing a small amount of lead wire was placed inside the ceramic work tube of a vertically orientated furnace (Carbolite TZF 15/50/610). The melted lead was evaporated at the centre of the furnace at a temperature of 830 °C. The metal vapour formed was carried out of the furnace by an inert nitrogen gas stream and diluted with a stream of air, during which the lead was oxidised to lead oxide. Both flow rates were set at 3 L/min by means of mass flow controllers (MFC). Resulting lead oxide nanoparticles were diluted in the second step by a stream of air (20 L/min) and used for whole-body inhalation experiments in exposure chambers.

### Characterisation of generated PbO nanoparticles

The distribution of nanoparticles (NPs) with respect to particle concentration was measured directly and continuously using a Scanning Mobility Particle Sizer (SMPS, model 3936 L72, TSI Inc., Shoreview, USA), with a size range of 8–230 nm (Additional file 1: Figure S1).

The long-term stability of PbO-NPs generation was high. The relative standard deviations in median particle diameter (25.8 nm) and total particle concentration (the first experiment: 1.23 × 10^6^ particles/cm^3^; the second experiment: 0.956 × 10^6^ particles/cm^3^) were 3.9% and 3.1%, respectively. These values indicate that both the generation of NPs and measurement by SMPS were reproducible.

Mass concentration of generated PbO nanoparticles was calculated by dividing the mass of PbO nanoparticles collected on the filter by the volume of air sample that was passed through the filter. Generated PbO nanoparticles were sampled on nitrocellulose filters (pore size 0.45 μm, diameter 25 mm, Millipore, Bedford, USA). Filters were dissolved in HNO_3_ using a UniClever microwave mineraliser (Plazmatronika, Wroclaw, Poland) and Pb content of the sample analysed by means of AAS (AAnalyst 600, PerkinElmer Inc., Shelton, CT, USA).

Surface area of the generated PbO-NPs (the first experiment: 4.30 × 10^3^ μm^2^/cm^3^; the second experiment: 4.21 × 10^3^ μm^2^/cm^3^) was calculated using the SMPS software [20].

Size and shape of generated PbO nanoparticles were determined by electron microscopy. Immediately after generation at the furnace output, PbO-NPs were collected by electrostatic precipitation using a Nanometer aerosol sampler (model 3089, TSI) on TEM grids (copper S160–4, 3 mm in diameter, 400 mesh grids, Agar Scientific, Electron Technology, Stansted, Essex, UK). The samples were analysed by means of a Magellan 400 L XHR microscope (FEI Company, Hillsboro, OR, USA), operating in the STEM mode. The PbO-NPs observed in the gas phase by SMPS were formed from agglomeration (size range approximately 40–50 nm) of primary 1–3 nm diameter particles (Additional file 1: Figure S2).

### Exposure to PbO nanoparticles

The inhalation chamber has been described in our previous study [21]. Adult female mice, with an average weight of approximately 24 g, were continuously exposed to lead oxide nanoparticles for 6 weeks (24 h/day, 7 days/week). Control animals were exposed to the same air as treated animals group without the addition of nanoparticles. Ten biological replicates (individuals) were used for each treatment. Two independent experiments were accomplished.

Animals in the experimental group (Pb) were exposed to an average concentration of 1.23 × 10^6^ particles/cm^3^; resp. 0.956 × 10^6^ particles/cm^3^ (mode 25.9 nm, geometric mean diameter 25.9 nm, median 25.8 nm, geometric standard deviation 1.66, average mass concentration 121.7 μg PbO/m^3^; resp. 149.3 μg PbO/m^3^). The estimated deposited dose of PbO over the 6-week inhalation period was 0.75 μg (resp. 0.92 μg in the second experiment) of PbO per gram of mouse body weight [22–24]. Detail calculation of deposited dose of PbO nanoparticles can be found in supplementary online material.

At the end of the exposure period, mice were sacrificed by cervical dislocation. The following organs were collected for chemical, histological and electron microscopic analyses: lung, brain, liver, kidney, and spleen.

### Histological analysis

For histological analysis, organs were fixed in 10% buffered neutral formaldehyde. Following overnight fixation in the fridge, samples were dehydrated using a series of increasing concentrations of ethanol, immersed in xylene and embedded in paraffin wax. Serial histological sections of 5 μm thickness were prepared and stained using standard Hematoxylin-Eosin staining. Selected sections were stained by Periodic Acid–Schiff (PAS) for detection of glycogen, by Green Trichrome for demonstration of connective tissue elements, especially collagen fibers, and by Luxol Fast Blue for visualization of myelin/myelinated axons in nerve tissue. Sections were examined by light microscopy (Leica DM5000 B, Leica Mikrosysteme GmbH, Vienna, Austria) in a blinded fashion. Photomicrographs were taken using a digital colour camera Leica DFC480 (Leica Mikrosysteme GmbH, Vienna, Austria).

### Immunohistochemistry

After deparaffinization and rehydration, sections were pre-treated in Target Retrieval Solution (pH = 9, DAKO, cat. No. S2367) for 20 min in a 97 °C water-bath. Non-specific secondary antibody binding was inhibited by incubation in a blocking serum for 20 min at room temperature (RT). Slides were incubated with the primary monoclonal antibody (PCNA, cat. No. 931143, full concentration, Invitrogen, USA; neurofilament, NF-M, cat. No. 2H3 DSHB, USA) for 1 h at RT. Secondary antibody was applied for 30 min at RT. The peroxidase-conjugated streptavidin-biotin system (Vectastain Elite ABC-Peroxidase kit, Vector Laboratories, USA) and chromogen substrate diaminobenzidine (DAB, cat. No. K3466, Dako, USA) were used for visualization. Sections were counterstained using Haematoxylin. A negative control was obtained by omitting the primary antibody from the labelling protocol.

### Detection of apoptotic cells

For the detection of apoptotic cells, the nuclear DNA fragmentation was labelled in situ by the terminal deoxynucleotidyl transferase-mediated dUTP nick end-labelling (TUNEL) method, according to the manufacturer’s protocol (ApopTag Peroxidase in situ Apoptosis Detection Kit; Chemicon, Temecula, CA, USA). Counterstaining with Haematoxylin was performed. A negative control was obtained by omitting the enzyme from the labelling protocol.

### Immunofluorescence

Following deparaffinization and rehydration, sections were pre-treated in Citrate Buffer (pH = 6) for 20 min in a 97 °C water-bath. Primary antibody (Na-K ATPase, cat. No. ab76020, Abcam, UK) was applied for 1 h at room temperature or overnight at 4 °C. Secondary antibody (goat anti-mouse Alexa Fluor® 488, cat. No. A-11001, Thermo Fisher, USA) was applied for 30 min at room temperature. Sections were washed in PBS and coverslipped in Prolong Gold anti-fade reagent containing DAPI (cat. No. P36935, Invitrogen, USA). The photos taken under a fluorescence microscope Leica DM LB2 (Leica Microsystems, Germany) were merged together in Adobe Photoshop 7.0 (Adobe, USA).

### Transmission electron microscopy

Samples of lungs, brain, liver, kidney and spleen were fixed in 3% glutaraldehyde for 24 h, washed three times in 0.1 M cacodylate buffer and post-fixed in 1% OsO_4_ solution for 1 h. Samples were then dehydrated in ethanol, followed by acetone and embedded in epoxy resin Durcupan. Semithin sections were stained with Toluidine Blue and examined by light microscopy in a blinded fashion. Ultra-thin sections (60 nm thick) were cut using an ultramicrotome Leica EM UC6 (Leica Mikrosysteme GmbH, Vienna, Austria) and placed on formvar-coated nickel grids.

Sections without further contrasting were observed using Morgagni™ 268 TEM (FEI Company, Eindhoven, Netherlands). Photographs were taken using a Veleta CCD camera (Olympus, Münster, Germany).

### Transmission electron microscopy – X-EDS

Elemental composition was determined by X-Energy Dispersive Spectroscopy (X-EDS). Ultra-thin sections were analysed on a TECNAI 120 (FEI Company, Eindhoven, Netherlands), equipped with a calibrated X-EDS Esprit2 (Bruker AXS, Madison, WI, USA). Working parameters were as follows: accelerating voltage of 120 keV with a LaB6 filament saturated with an emission of 3 (scale of 6); condenser aperture of 100 μm apparent diameter without objective diaphragm. As the TEM mode the Nanoprobe one was used allowing a beam size of 70–100 nm. The sample was tilted at 15° solid angle to achieve a take-off angle of 45°. Counting times were a minimum of 120 s, up to 200 s, with a dead-time between 5% and 20%. These parameters optimised the analytical time regarding the degradation of the sample by the electron beam.

### Analysis of Pb content in mouse organs

The weights of individual organs were determined and the values were recorded for later quantitative evaluation. Organ samples were digested in 3 ml of concentrated nitric acid (sub-boil grade; produced using a quartz distillation system model MSBQ 2, Maasen, Eningen, Germany) by microwave-assisted digestion. Samples were completely decomposed in pre-cleaned PTFE vessels in a closed, pressurised autoclave system (UniClever, Plazmatronika, Wroclaw, Poland). The digestion programme consisted of three 5-min steps, involving application of 70%, 90% and 100% microwave power (100 W), controlling working pressure within limits of 35/32, 40/35 and 45/42 bar, respectively. After a 10-min cooling period, digests were quantitatively transferred to pre-cleaned PP vials, diluted and adjusted to a final mass of 10 g with ultrapure water (Ultra Clear system, SB Barsbüttel, Germany). Blank samples (typically *n* = 12 per sampling series) were simultaneously processed in the same way.

Lead content of digests was determined by electrothermal atomic absorption spectrometry (ET AAS), employing AAnalyst 600 Perkin-Elmer (PerkinElmer Inc., Shelton, CT, USA) instrumentation according to the recommended procedure. A mixture of ammonium phosphate and magnesium nitrate was used as a combined chemical modifier. A method of standard addition calibration was applied for quantitation. Lead content of the following organs was determined at the end of week 6 of treatment: lungs, liver, kidney, brain and spleen.

### Analysis of mouse blood for Pb content

Blood samples were collected by cardiac puncture into 1 ml plastic Eppendorf tubes containing a small amount of heparin. Whole blood was divided into three fractions by centrifugation and proteins were further separated from the remaining supernatant using methanol (300 μl). Samples were stored at 5 °C for subsequent analysis.

Individual fractions of blood were decomposed by means of an ozone and NO_x_ nitrous oxides gas mixture employing the system Dry Mineralizer Apion (Tessek, Prague, Czech Republic) under atmospheric pressure. Blood samples were quantitatively transferred to pre-cleaned glass vials and 1 ml of 1 M nitric acid (high purity sub-boil grade) was added to each sample for enhanced mineralisation. Vials were embedded in a thermo-block heating system in which samples were first dehydrated at 110 °C (ramp time 45 min) for 60 min, then mineralised at 380 °C (ramp time 4 h) for a period 8 h with simultaneous introduction of the gas mixture. After cooling of vials, digest samples were diluted in 1 ml 1 M nitric acid (sub-boil purity), quantitatively transferred to plastic scintillation vials, adjusted with ultrapure water (Ultra Clear system, SB Water Systems, Barsbüttel, Germany) to a final mass of 3 g and stored for subsequent analysis. Blank samples were simultaneously processed in the same manner.

The content of lead in blood digests was determined by electrothermal atomic absorption spectrometry (ETAAS) employing AAnalyst 600 (PerkinElmer Inc., Shelton, CT, USA) instrumentation according to recommended procedures. A mixture of ammonium phosphate and magnesium nitrate was used as a combined chemical modifier. A method of standard addition calibration was applied for quantitation. Lead content was determined at the end of week 6 of exposure.

### Data analysis

Results were reported as the mean value ± standard deviation. Data were analysed using Statistica 8.0 software (StatSoft. Inc., Tulsa, OK, USA). Student t-test was used to determine differences between experimental and control groups. Values of *p <* 0.05 were considered to be statistically significant.

## Results

### Content of lead in organs and blood following six week exposure to PbO nanoparticles

**Fig. 1:**
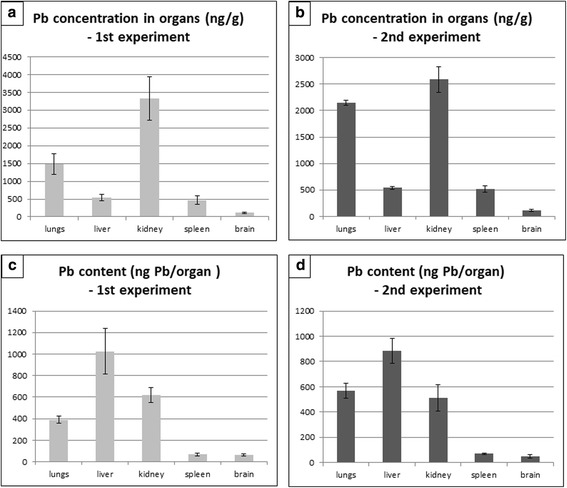
Content of lead in organs following 6 weeks exposure of PbO-NPs. **a, b** Concentration of lead in organs following six weeks exposure. **c, d** Lead content (uptake) in organs following six weeks exposure

The content of lead in the organ of entry (lung) as well as in the secondary organs was markedly increased after six weeks of nanoparticle inhalation compared to controls (Fig. 1a, b). The highest concentration of lead was found in the kidney, followed by the lungs, liver and spleen, and finally the brain (Fig. 1a, b). The lead concentration in organs of mice exposed to PbO nanoparticles was more than 1500 times higher in lungs, 140 times in kidney, 140 times in liver, 470 times in spleen and about 120 times higher in brain in comparison to the control organs (Additional file 1: Table S1). Similar trend was observed in the second independent experiment (Additional file 1: Table S2).

We also compared lead mass (uptake) after the treatment in all organs that were investigated (Fig. 1c, d). This analysis showed the highest amount of lead to be in the liver, followed by kidney, lungs, and similar lead content was found in spleen and brain.

**Fig. 2:**
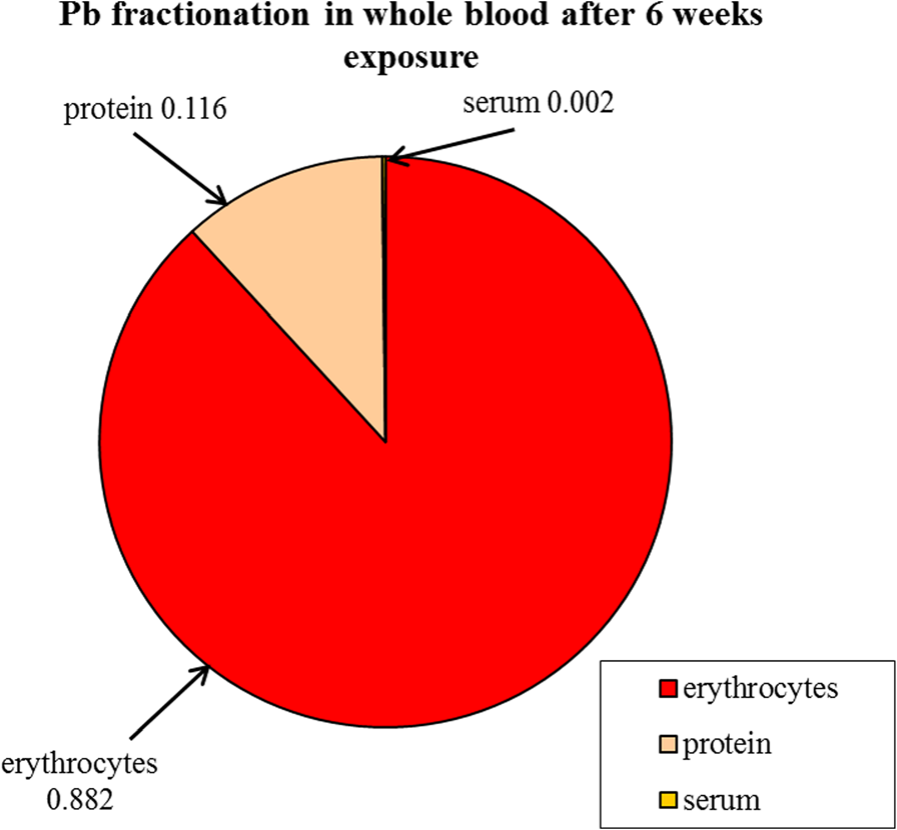
Content of lead in blood following 6 weeks exposure

Moreover, we determined the amount of lead in three fractions of blood (Fig. 2). The largest proportion of lead was found in blood cell fraction (88.2% of lead), lower proportion in the plasma protein fraction (11.6% of lead) and a negligible proportion in serum (below LOD, representing value of about 0.2% of total lead concentration in whole blood). The concentration of lead in whole blood of mice exposed for six weeks to PbO was 132 ng·g^−1^, whereas this was below the detection limit of 11 ng·g^−1^ in the control animals.

### Inhalation of lead oxide nanoparticles exert multiple negative effects on mouse lungs as a primary target organ

**Fig. 3:**
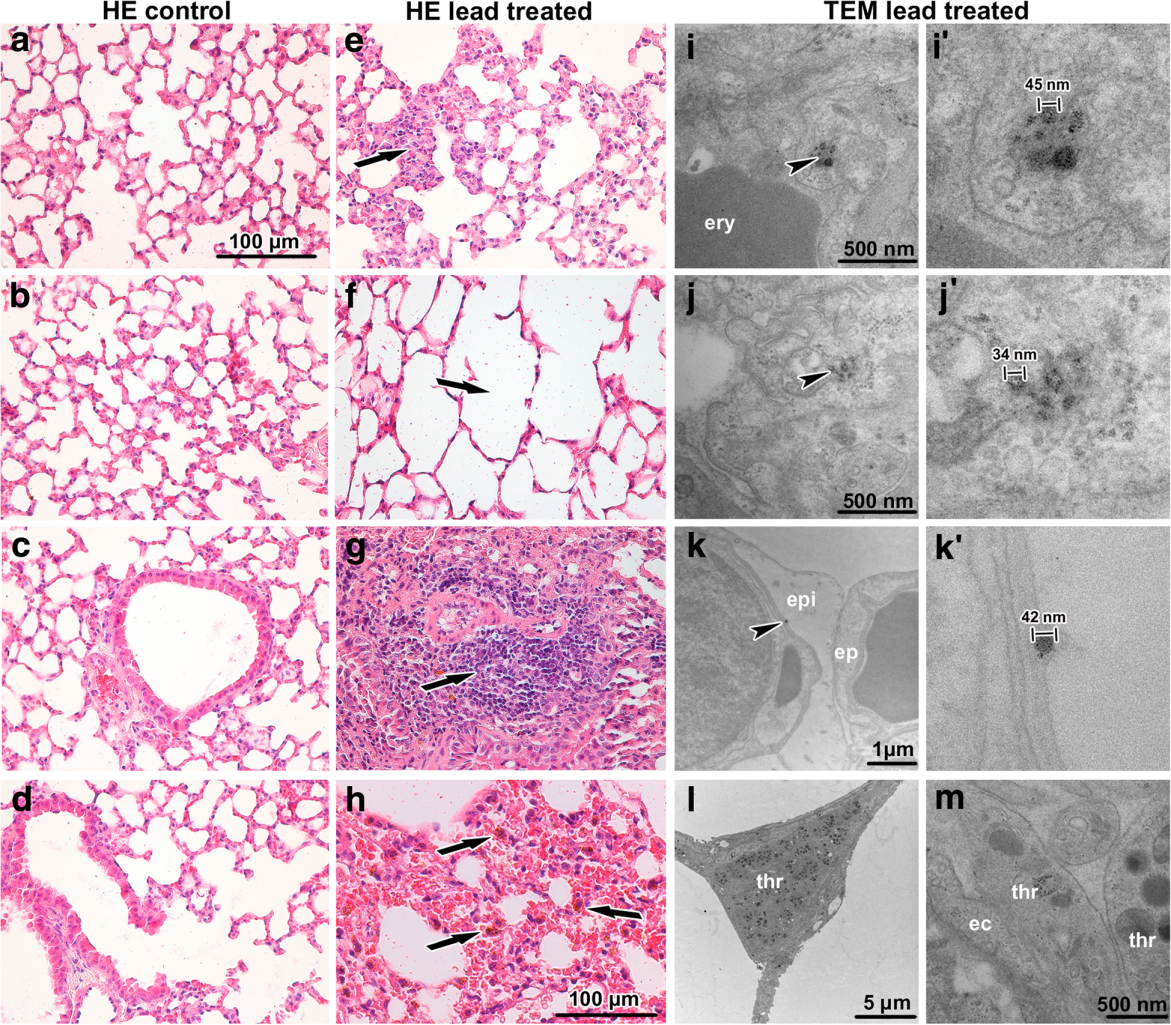
Effect of inhalation of lead oxide nanoparticles on lung following 6 weeks exposure. **a**-**d** control tissues stained with Hematoxylin-Eosin. **e**-**h** exposed tissues stained with Hematoxylin-Eosin. Arrows show alterations in lung tissue as thickened septs with congested capillaries (**e**), alveolar emphysema (**f**), full-range perivascular infiltrate (**g**), hemostase with siderophages (**h**). Scale bar in panels **a**-**h** = 100 μm. **i**-**m** lung tissue after treatment in transmission electron microscope. **i**, **i**’ nanoparticles in cytoplasmic vesicle of alveolar epithelial cell type I, erythrocyte (ery) present in alveolar space. **j**, **j**’ nanoparticles in vesicle of alveolar epithelial cell type I. **k**, **k**’ nanoparticle beneath edematous changed alveolar epithelial cell type I (epi), neighboring alveolar epithelial cell type I (ep) is intact. **l** microthrombus in lung venule, accumulation of thrombocytes (thr) is obvious. **m** thrombocyte (thr) adhering to endothelial cell (ec). Arrowheads show nanoparticles

**Fig. 4:**
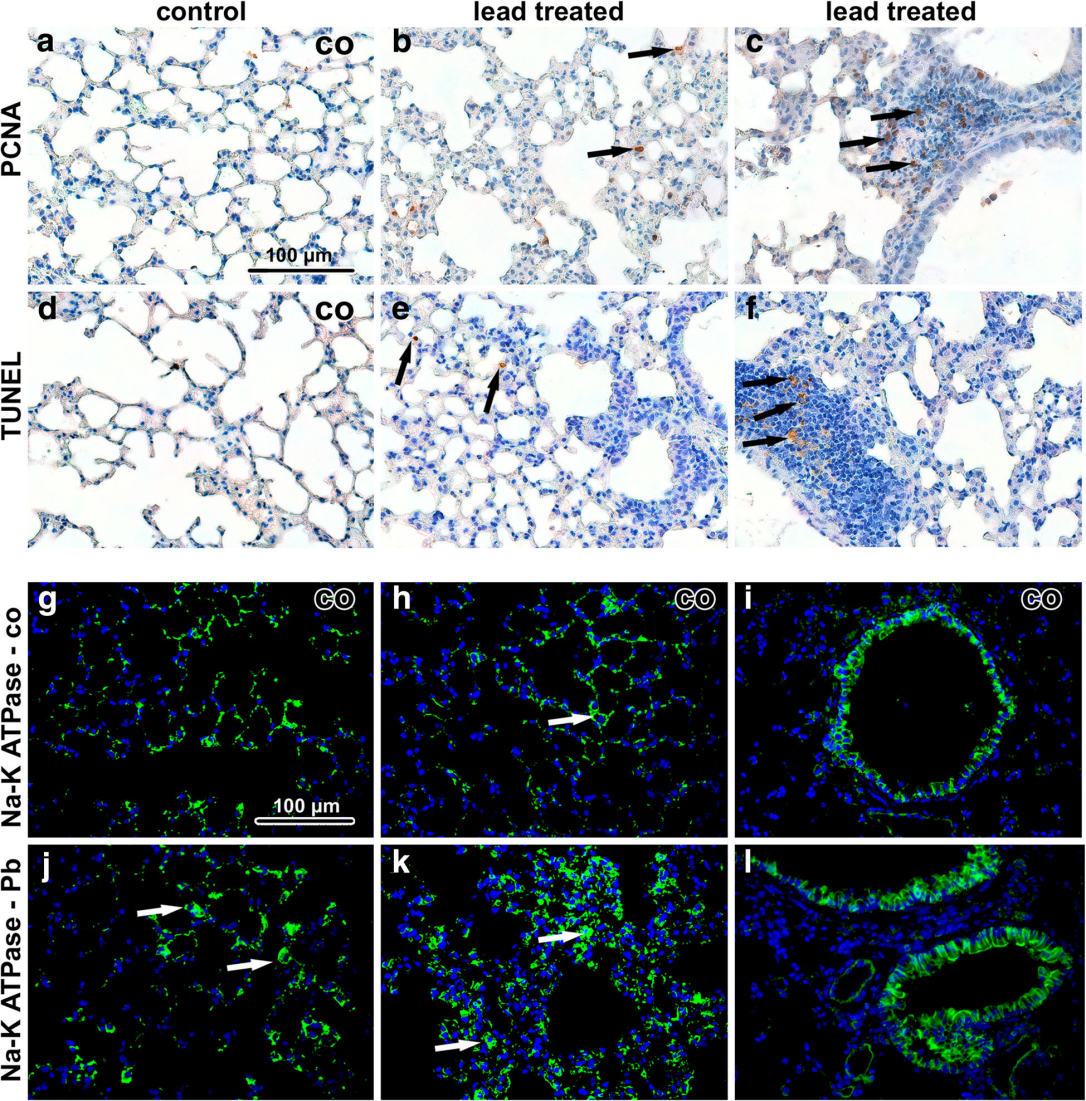
Immunostaining of lung samples following 6 weeks exposure to PbO-NPs. **a**-**c** PCNA in lung tissue. Arrows indicate PCNA-positive cells in lung. The highest amount of positive cells is in infiltrates. **d**-**f** TUNEL in lung tissue. Arrows indicate TUNEL-positive cells, mainly macrophages and some immune cells in infiltrates. **g**-**i** Na-K ATPase in control samples. Cells with high positivity are macrophages and bronchiolar epithelial cells. **j**-**l** Na-K ATPase in treated samples without outstanding changes. Arrows indicate positive macrophages. Scale bar in all panels = 100 μm

Histopathological analysis revealed that inhalation of lead oxide nanoparticles caused severe alterations in lung morphology following six weeks exposure. Lungs of these animals exhibited moderate hyperaemia, congested capillaries, enlarged pulmonary septa, haemostasis in basal parts of lobes with presence of many siderophages (macrophages with pigment hemosiderin) and the presence of alveolar emphysema in some areas of the lungs (Fig. 3). Increased numbers of neutrophils and macrophages were found in the alveoli. Bronchioles exhibited the presence of increased mucous secretion, desquamated epithelial cells, neutrophils and sporadic red blood cells. Inhalation of PbO nanoparticles caused also extensive perivascular and peribronchiolar lymphocyte infiltration, which are typical of sub-chronic inflammation. We observed a large number of TUNEL-positive cells in pulmonary basal lobes and in lymphocyte infiltrates (Fig. 4). In addition, elevated numbers of proliferating cells were found in infiltrates, indicating intensive reparative processes initiated in these areas. Activity of Na-K ATPase in lung alveolar tissue was not significantly altered after exposure to PbO nanoparticles (Fig. 4).

Examination using transmission electron microscopy (TEM) demonstrated oedematous enlargement of alveolar epithelial type I (PI) cells and endothelial cells in some regions of the lungs (Fig. 3i-m). Fragmentation of alveolar epithelial type I cells and endothelial cells of the capillaries with denuded basement membranes was also detected. Moreover, the occurrence of apoptotic and necrotic macrophages and granular pneumocytes was observed.

Nanoparticles were located both in alveolar cavities and within cells (Fig. 3i-k’), mainly in alveolar epithelial type I cells (membranous pneumocytes, PI) but not in alveolar epithelial type II cells (granular pneumocytes, PII). Furthermore, they were also found in phagosomes of lung macrophages and neutrophils. In the alveolar cavities, nanoparticles were enveloped in surfactant. Nanoparticles formed clusters within cytoplasmic vesicles. The contents of the vesicles, completely enveloped by cytoplasmic membrane, were more electron-dense than the surrounding cell cytoplasm. Nanoparticles in the vesicles exhibited different sizes, they were well-separated from each other and often located adjacent to the vesicular membrane. Notably, increased amount of tubular myelin (an intermediate step in the formation of surface film) was indicative of enhanced production of surfactant by alveolar epithelial type II cells (PII). Moreover, we observed frequent incidence of thromboses in capillaries and venules (Fig. 3l, m). There were no nanoparticles found in the lungs of control mice exposed to clean air.

### Hypertrophy of hepatocytes and steatosis of liver was observed following the inhalation of lead oxide nanoparticles

**Fig. 5:**
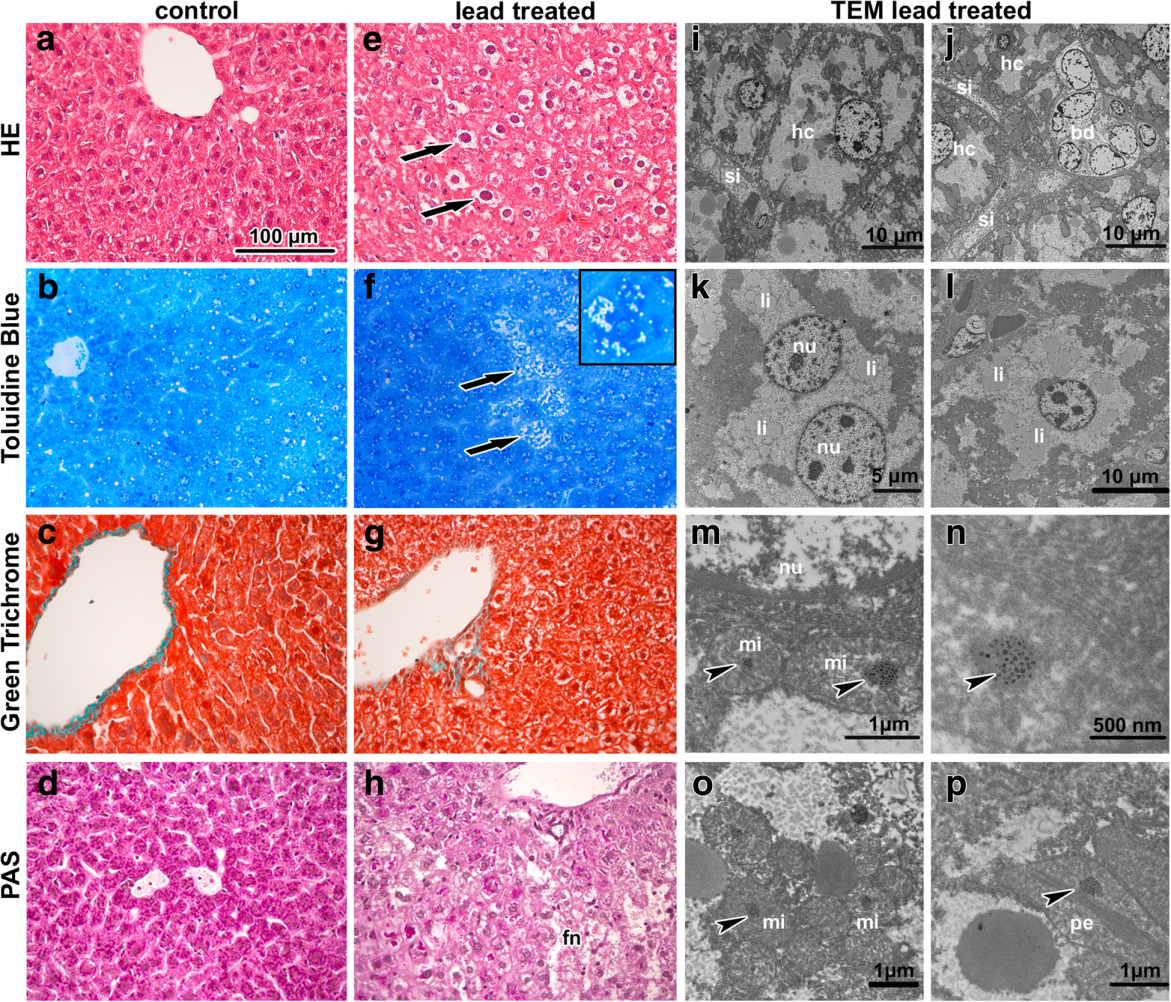
Effect of inhalation of lead oxide nanoparticles on liver following 6 weeks exposure. **a, e** Hematoxylin-Eosin stained liver samples. **b, f** Toluidine Blue stained semithin sections. **c, g** Green-Trichrome stained liver samples. **d, h** PAS staining. Arrows show alterations in liver tissue as hypertrophy of hepatocytes with karyomegaly, vacuolated cytoplasm (**e, f**), and focal necrosis (fn). Detail of groups of lipid droplets in hepatocyte identifiably in Toluidine Blue staining (the upper corner in panel **f**). Scale bar in panels **a**-**h** = 100 μm. **i-p** Subcellular changes in liver tissue after lead treatment. **i, j** affected hepatocytes (hc) with vacuolated cytoplasm, unnatural sinusoidal endothelial cells (si), and bile duct epithelial cells (bd). **k, l** abundant lipid droplets (li) and nuclei (nu) in hepatocyte. **m-o** nanoparticles (arrowheads) in hepatocyte mitochondria (mi), next to nucleus (nu). **p** nanoparticles (arrowhead) in hepatocyte peroxisome (pe)

Following sub-chronic exposure to PbO nanoparticles, significant changes occurred in liver as a secondary target organ (Fig. 5), mainly manifested by hepatocyte enlargement and hydropic degeneration. Hypetrophy of hepatocytes was combined with karyomegaly, mostly located in centrilobular regions, and with increased amount of binucleated hepatocytes. We found occasional periportal inflammation in some areas, predominantly lymphocytic infiltration. In some samples, areas of hepatic necrosis were observed. PAS staining confirmed impaired glycogen content in hepatocytes (Fig. 5 d, h). There was no evidence of granuloma, noticeable sinusoidal dilatation, hepatic fibrosis (Fig. 5c, g) or bile duct abnormalities.

When examined by TEM, overall morphology of hepatocytes around the central vein was almost same in the animals exposed to nanoparticles as in controls (Fig. 5i-p). Nuclei (1 or 2) were round, small, with smooth contours, with prevalent euchromatin and with few nucleoli. Periportal hepatocytes (zone 1 of liver acinus) instead exhibited typical signs of necrosis. Cells in this area were enlarged with obvious cellular damage, they exhibited clear round nuclei, cytoplasm appeared almost empty and cell organelles were degraded or showed severe pathological signs including hydropic dystrophy (membrane rupture, presence of vacuoles in their bodies and oedema). Periportal hepatocytes are in the most intimate contact with the arterioles, thus representing the best oxygenated hepatocytes and the first group of cells, which absorb blood-borne lead oxide nanoparticles or lead in non-particulate form.

Mitochondria in affected hepatocytes were not evenly distributed throughout the cytoplasm as observed in the controls but they were accumulated around the nucleus and peripherally underneath the plasma membrane. In some cases, they formed short radial septs inside the hepatocytes. Amount of mitochondria exhibiting degeneration of their inner membranes was insignificant. Cisternae of granular endoplasmic reticulum were found in direct contact with mitochondria. In PbO-treated animals, secondary lysosomes (hard upon bile canaliculus) and sporadic peroxisomes were individually dispersed. Lysosomes occurred in the cytoplasm of PbO-treated animals to approximately the same extent as in the controls.

Numerous lipid droplets (tens in a single hepatocyte), resembling electron-dense vacuoles of different sizes, were accumulated in the cytoplasm of hepatocytes (Fig. 5k, l). They either formed groups of lipid droplets or they were located individually with a size ranging from 0.5 to 3 μm. Such accumulation of small intracytoplasmic fat vacuoles (liposomes) is indicative of microvesicular steatosis of liver.

Epithelial cells of the bile ducts were also affected following the exposure to lead oxide nanoparticles. Nuclei and cytoplasm of the lining cells exhibited signs of necrosis. The damage of hepatic endothelial cells was about the same as in bile duct epithelia. In contrast, the ultrastructure of Kupffer cells and Ito cells did not display any changes. Also the numbers of macrophages and Ito cells were unaffected compared to the controls.

Following six weeks of inhalation, nanoparticles were found inside hepatocytes (Fig. 5m-p) where aggregates of nanoparticles accumulated within the centres of their mitochondria. These aggregates were all of about the same size (diameter of about 20–30 nm), they were enveloped by an electron-dense mitochondrial matrix and formed clusters in number of up to tens inside one mitochondrion (Fig. 5m-o). Thus, nanoparticles exhibited different morphology in liver in comparison to the original nanoparticles in the alveolar epithelial cells I. Also their aggregates in livers were smaller and more uniform. Nanoparticles were also found in peroxisomes of hepatocytes possessing the same features as those in mitochondria and they were freely located within the hepatocyte cytoplasm (Fig. 5p). In contrast, nanoparticles were not found in Kupffer cells, Ito cells and bile ducts.

**Fig. 6:**
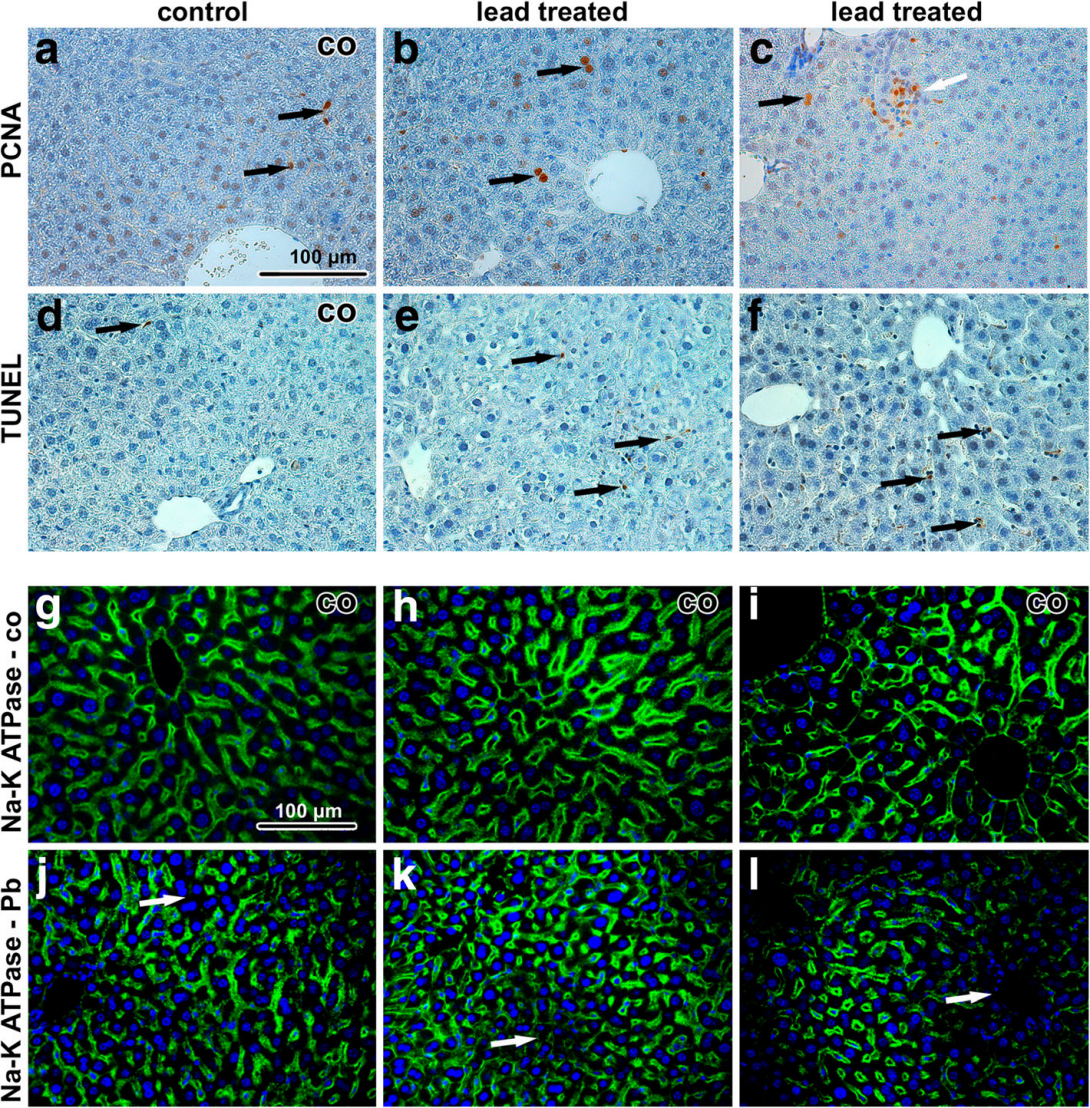
Immunostaining of liver samples following 6 weeks exposure to PbO-NPs. **a-c** PCNA in liver tissue. Arrows indicate PCNA-positive cells in liver**.** In controls, PCNA positive cells are mainly sinusoid lining cells while after treatment (**a**), PCNA positive are hepatocytes (**b, c** – black arrows). The highest amount of positive cells is present in mononuclear infiltrates (islands of extramedullary hematopoiesis, **c** - white arrow). **d-f** TUNEL in liver tissue. Arrows indicate TUNEL-positive cells, mainly sinusoid lining cells as e.g. Kupffer cells. **g-i** Na-K ATPase in control samples. Membranes of hepatocytes show high positivity of enzymes, cords of hepatocytes are commonly arranged in lobules. **j-l** Na-K ATPase in treated samples. White arrows indicate regions in lobules without positivity. Hepatocytes are irregularly organized, different sized and often binucleated. Scale bar in all panels = 100 μm

Interestingly, the number of apoptotic cells (TUNEL-positive) in organs of animals exposed to PbO NPs was unchanged in comparison to the controls (Fig. 6). In both, mainly Kupffer cells were TUNEL-positive. Some Kupffer cells also expressed proliferating cell nuclear antigen (PCNA), with their numbers being about the same in PbO-exposed and control animals. In contrast, PCNA-positive hepatocytes (mainly binucleated) were more frequent in Pb-exposed animals compared to the controls. Expression of Na-K ATPase unravealed alterations to functioning of hepatocytes and to sinusoid arrangement. Specifically, activity of Na-K ATPase was almost vanished in some areas of liver lobules following exposure to PbO nanoparticles (Fig. 6).

### X-EDS analysis verified the presence of lead in liver ultrathin sections

**Fig. 7:**
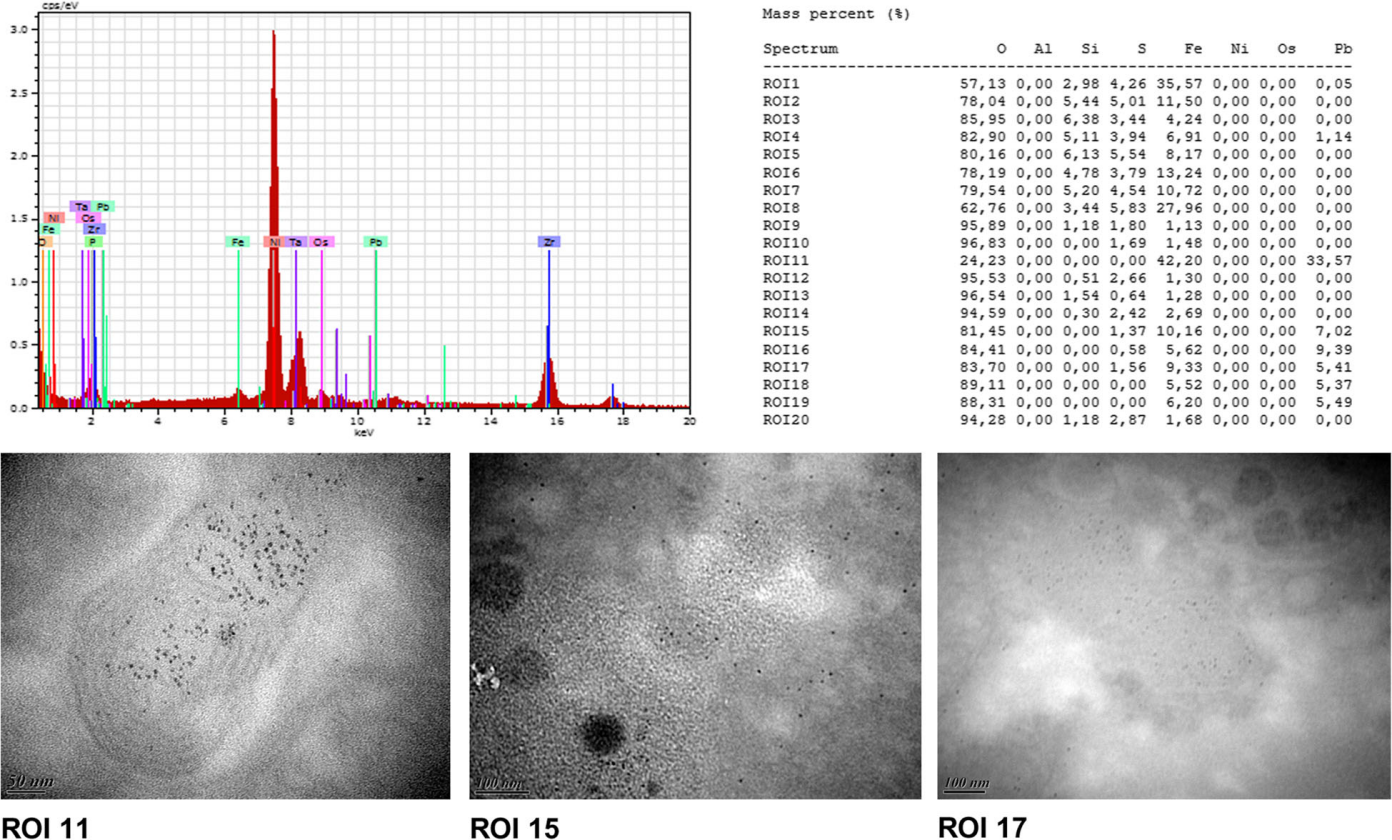
X-EDS analysis in treated samples of liver. Data obtained from analyzed ROI of treated samples. Nickel observed during analysis was issued from the grid

Chemical composition of livers was analysed by X-EDS to verify lead content of nanoparticles that were visible on ultrathin sections (Fig. 7). Approximately 20 different Region Of Interest (ROI) were analysed. The analysis confirmed the presence of lead in treated samples and no Pb in control samples. Lead was accumulated in the cells and Pb peaks were detected very clearly.

### Minor effect on mouse kidney was observed following inhalation of lead oxide nanoparticles

**Fig. 8:**
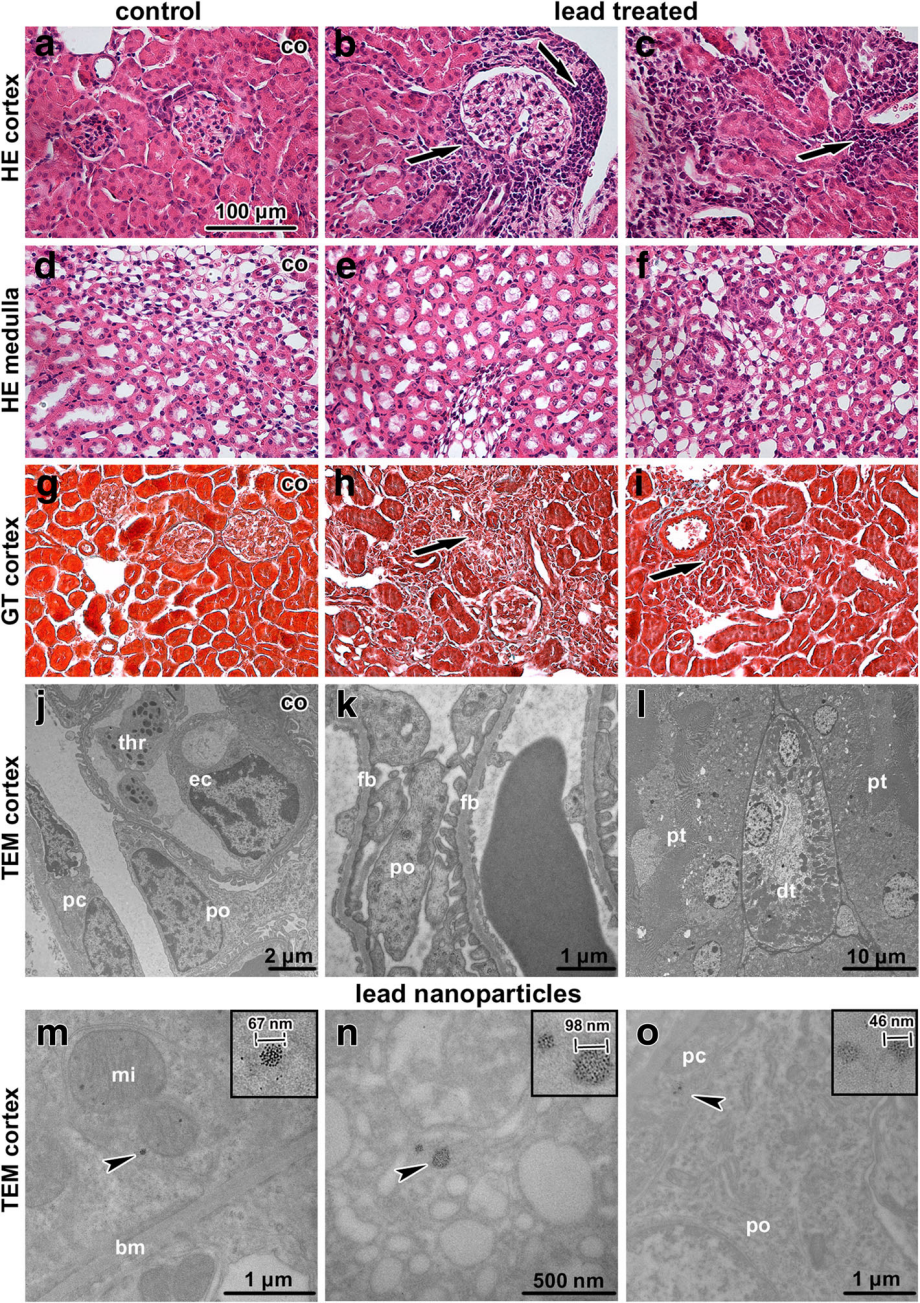
Effect of inhalation of lead oxide nanoparticles on kidney following 6 weeks exposure. **a-f** kidney tissues stained with Hematoxylin-Eosin. **g-i** kidney tissues stained with Green-Trichrome. Arrows indicate mononuclear infiltrations. Scale bar in panels **a**-**i** = 100 μm. **j-o** kidney tissue in transmission electron microscope. **j** control sample of renal corpuscle, podocyte (po), parietal cell (pc), endothelial cell (ec), thrombocyte (thr) in glomerular capillary. **k** alteration of filtration barrier (fb) morphology after treatment, body of podocyte (po). **l** intact tubules - proximal tubules (pt), distal tubule (dt) in kidney cortex of lead treated animal. **m** nanoparticle freely in epithelial cell cytoplasm of proximal tubule, mitochondrion (mi), basement membrane (bm). **n** nanoparticles freely in intercalated cell cytoplasm of cortical collecting duct. **o** nanoparticles freely in parietal cell cytoplasm (pc) of Bowman capsule, next to podocyte (po). Details of nanoparticles in the upper corner of panels **m**, **n**, **o**. Arrowheads show nanoparticles

Histopathological analysis revealed that inhalation of lead oxide nanoparticles caused only mild alterations in kidney morphology after sub-chronic exposure (Fig. 8). In some samples, we observed areas with inflammatory infiltrates around renal corpuscles, around renal tubules and blood vessels in the renal cortex. Similar mononuclear cells infiltrates were occasionally found also in control animals. Affected renal corpuscles were slightly enlarged with dilated glomerular capillaries in comparison to the control tissues. The other types of pathological microscopic changes in kidney were rare (Additional file 1 **:** Table S3, Table S4).

Ultrastructural analysis of kidney tissue unravelled only minimal morphological changes following nanoparticle treatment. Renal tubules showed normal appearance (Fig. 8l), and cortical and medullary collecting ducts had normal structure. Some apoptotic cells were observed in tubules; however, their amount was similar to the controls.

We observed moderate differences in the arrangement of filtration barrier in kidneys of PbO-treated animals. Both laminae rarae were filled by electron-dense material (fine deposits), with lamina densa being thicker compared to the controls. The typical electron contrast among these three layers (laminae) disappeared after lead exposure (Fig. 8k). The filtration membrane was diffusely thickened with intramembranous electron-dense deposits. The distance between the cytoplasmic membrane of endothelial cells and the cytoplasmic membrane of podocytes in the control kidneys was approximately 140–150 nm. Upon exposure to PbO this distance increased to more than 200 nm.

Lead oxide nanoparticles were accumulated in the epithelial cells of the proximal tubules in kidney cortex, the parietal cells of Bowman’s capsule and in the epithelial cells of the cortical collecting ducts (Fig. 8m-o). These findings demonstrate the ability of lead oxide nanoparticles to pass through epithelial cells in various parts of the kidney. Lead oxide nanoparticles were dispersed in the cytoplasm but not within cell organelles. No particles were observed inside the nuclei of kidney cells.

**Fig. 9:**
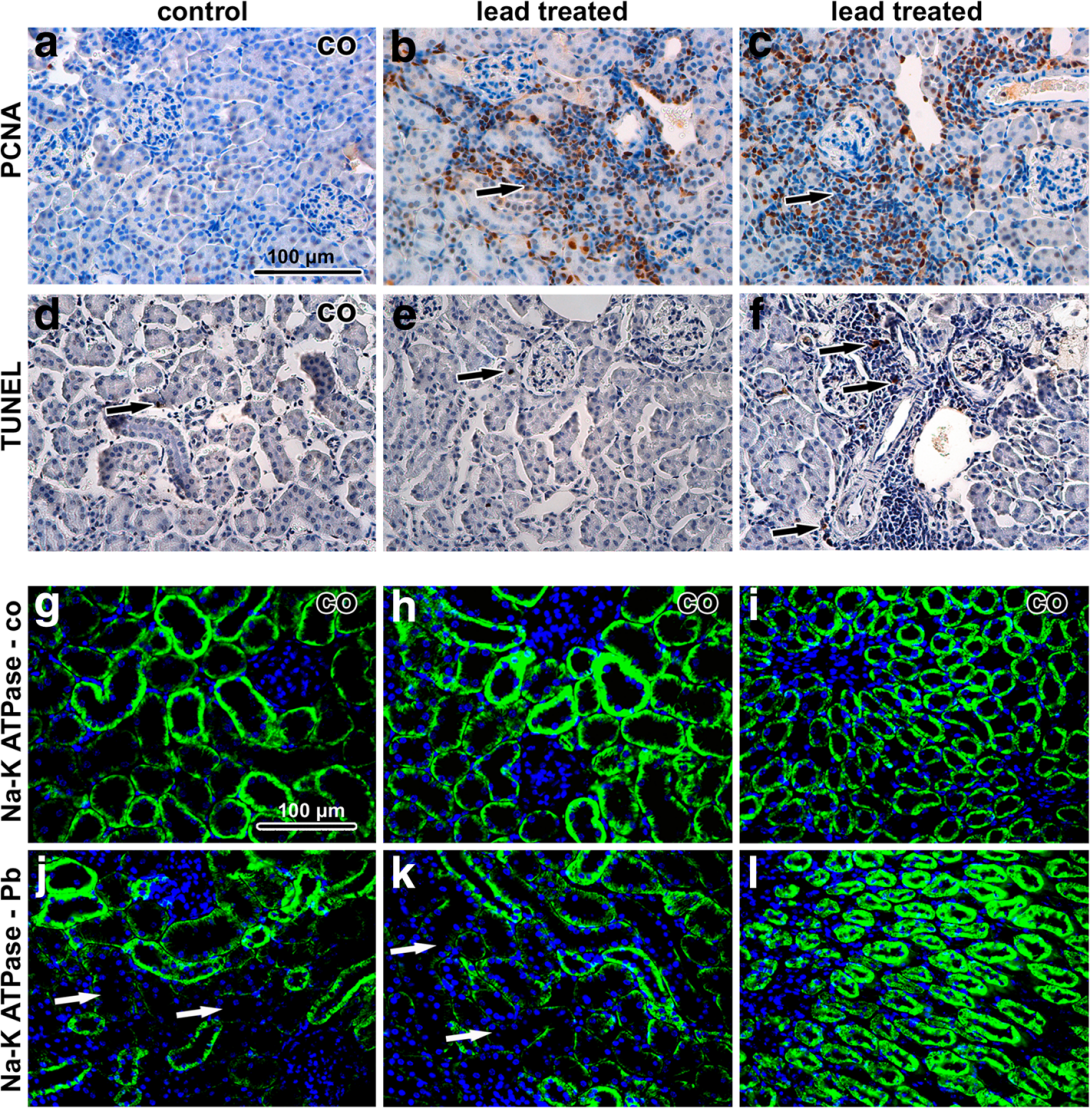
Immunostaining of kidney samples following 6 weeks exposure to PbO-NPs. **a-c** PCNA in kidney tissue. Arrows indicate PCNA-positive cells in kidney. An enormous amount of positive cells is in infiltrates. **d-f** TUNEL in kidney tissue. Arrows indicate TUNEL-positive cells, individually located mostly in infiltrates. **g-i** Na-K ATPase in control samples. Cells with high positivity of enzyme are epithelial cells of distal tubules. **j-l** Na-K ATPase in treated samples. Arrows indicate decreased activity of enzyme in the epithelial cells of proximal tubules. Scale bar in all panels = 100 μm

Exposure to PbO nanoparticles caused increase in the number of TUNEL-positive cells present in lymphocyte infiltrates (Fig. 9). In other regions of the kidney, TUNEL-positive cells were only rare. In addition, inflammatory infiltrates contained large numbers of proliferating (PCNA-positive) immune cells. Activity of Na-K ATPase was decreased mainly in cells of proximal tubules of animals exposed to PbO nanoparticles.

### Inhalation of lead oxide nanoparticles did not produce pathological changes in mouse spleen

We did not observe any significant pathological changes in the spleen as a secondary target organ following inhalation of lead oxide nanoparticles (Additional file 1: Figure S5). Animals exposed to PbO nanoparticles exhibited typical spleen morphology undistinguishable from the controls. An arrangement of red pulp and white pulp with blood vessel supply was normal. Areas of erythroblastic islands and regions of granulopoiesis were visible, similar to the controls. The only exception was an increased incidence of megakaryoblasts and megakaryocytes in the pulp of PbO-treated animals (Additional file 1: Figure S5 C, D).

Occurrence of individual lead oxide nanoparticles in spleen sections was rare and these were distributed throughout the cell cytoplasm. Nanoparticles were located in macrophages, erythroblastic cells and reticulocytes (Additional file 1: Figure S5 g, h).

### Inhalation of lead oxide nanoparticles caused changes in the hippocampal area of the mouse brain

**Fig. 10:**
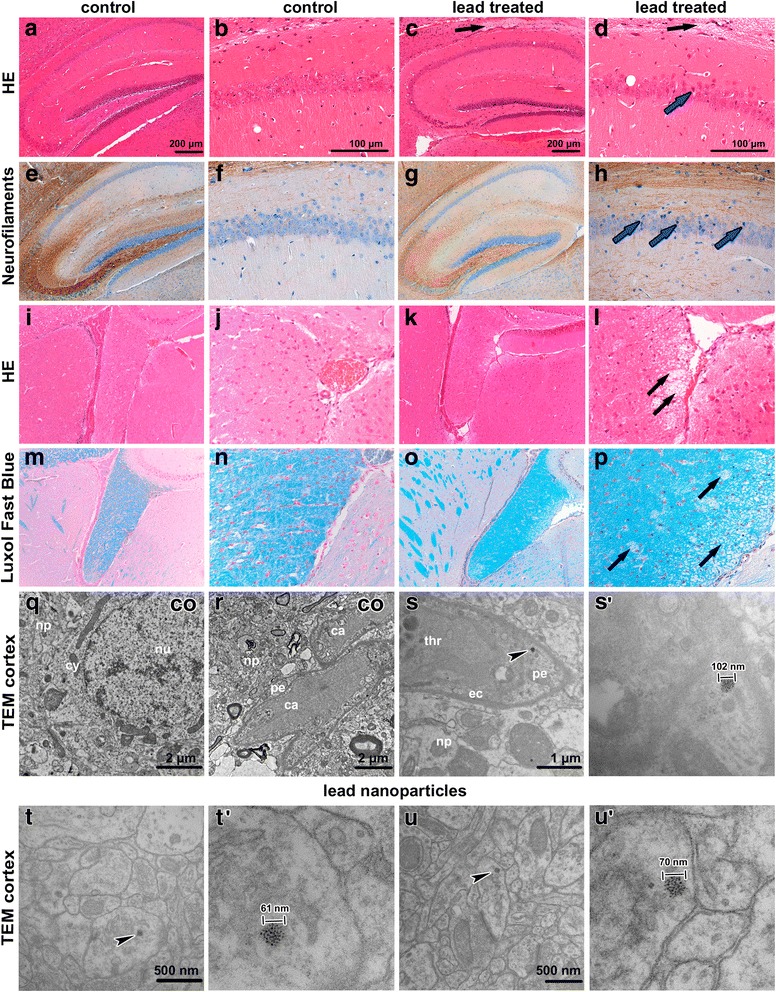
Effect of inhalation of lead oxide nanoparticles on brain following 6 weeks exposure. **a**, **b** HE staining - hippocampal region in control (co) sample, round and palely stained nuclei of pyramidal neurons of Ammon’s horn in CA1 region. **c**, **d** HE staining **-** hippocampal region in treated sample. Black arrows (**c**, **d**) show region of spongiform changes in white matter. **e**-**h** Neurofilament staining of hippocampal region with affected neurons in CA1 region. Blue arrows indicate dark shrunken damaged pyramidal neurons (**d**, **h**). **i**-**l** HE staining of fiber tracts beneath hippocampal region. **m**-**p** Luxol fast blue staining. Black arrows (**l**, **p**) point to regions of spongiform changes in white matter. Scale bar in panels **a**, **c**, **e**, **g**, **i**, **k**, **m**, **o** = 200 μm. Scale bar in panels **b**, **d**, **f**, **h**, **j**, **l**, **n**, *p* = 100 μm. **q**-**u**’ Subcellular analysis of brain following 6 weeks exposure to lead oxide nanoparticles. **q**, **r** brain tissue in control sample with neuron nucleus (nu), its cytoplasm (cy), and surrounding neuropil (np), capillaries (ca) with continuous endothelial lining, thick basal lamina, pericyte (pe), and surrounding neuropil (np). **s** brain capillary after treatment, thrombocyte (thr) in capillary lumen, endothelial cell (ec) is unaffected, pericyte cytoplasm (pe) contains nanoparticle, neuropil (np) is surrounding. **s**’ detail of nanoparticle in pericyte. **t**, **t**’ nanoparticle observed freely in neuron process. **u**, **u**’ presynaptic (pre) terminal with synaptic vesicles and mitochondrion, and postsynaptic (po) terminal with observed nanoparticle. Arrowheads show nanoparticles

Exposure to lead oxide nanoparticles caused changes (Fig. 10) in the hippocampal region. We observed dark, shrunken, damaged neurons in the pyramidal layer of Ammon’s horn, predominantly in CA1 region (Fig. 10d, h). Tracts of nerve fibers surrounding the hippocampal area were also affected. Luxol Fast Blue staining confirmed spongiform changes in these regions (Fig. 10c, d, l, p). Neither the number of PCNA-positive neurons (Additional file 1: Figure S6) nor TUNEL-positive cells was changed after the exposure (data not shown).

In brain tissue, lead oxide nanoparticles were numerous, in contrast to the kidney or spleen (Fig. 10s-u’). The particles were located in cytoplasm of neurons, in their processes and they were also closely associated with synapse regions. Moreover, they were found in pericytes (mural contractile cells of capillaries). Lead oxide nanoparticles were dispersed throughout the cytoplasm or grouped in cytoplasmic vesicles and they exhibited similar appearance to those in the lungs or kidneys.

## Discussion

Currently, the routes of exposure to nanoparticles are numerous and they depend on the conditions related to how and where the nanoparticles are generated and/or employed, e.g. factories, cosmetics, sprays, food, etc. Therefore, same nanoparticles can enter the body by different routes depending on the process of their production. Still, the respiratory tract is the main and typical route by which nanoparticles (NPs) enter the human body [25–27]. This route of entry may continue by multiple secondary distribution avenues, including olfactory path to the brain, translocation to the blood or entry to the GI system during lung clearance.

The lung represents an organ with maximum exposure to PbO nanoparticles and susceptibility to any toxic effects. As macrophages have difficulties identifying such small particles and carrying out phagocytosis, lung defence against nanoparticles is very weak [28]. Nanoparticles are absorbed and deposited in epithelial cells of bronchioles and alveoli, they may rapidly cross the barrier and get absorbed into the circulation, and then migrate to secondary target organs such as the liver and kidney [25, 29]. In agreement with these findings, our analysis revealed increased amount of lead, not just in the primary organ (lung), but also accumulations in the secondary organs. After six weeks of exposure to PbO, the highest concentration of lead was found in the kidney, followed by the lungs, liver, spleen and brain. The same results were previously reported for the treatment with low doses of PbS nanoparticles (kidney > lung > liver) [30]. However, higher doses of PbS nanoparticles resulted in increased amount of lead in the liver compared to the lungs (kidney > liver > lung). Thus, distribution of nanoparticles within the body can be influenced not only by nanoparticle properties but also their dose.

Previous studies revealed large accumulation of lead in the skeleton [2] at balanced conditions. As we used sub-chronic time point, we can predict that steady condition was not established yet and lead content in bone will not be so high. This is also supported three-compartment model of human lead metabolism postulating that from blood to bone is transported approximately 15% of lead from lead daily intake [31]. Secondary target organs therefore contain proportionally larger amount of total lead in our experimental conditions before their exclusion by urine/feces or their accumulation in skeleton.

However, it is necessary to mention that not all lead found in our target organs is in nanoparticles form. Some nanoparticles can be dissolved after in vivo deposition in the lungs, leading to ions formation, and these ions can be released and transported from the site of their production to other target organs resulting in continual dissolution (and thus ion generation) of the residual nanoparticles [32]. Moreover, the dissolution rate of nanoparticles is not a constant factor but depends on particle size, coating, stability, manufacturing process, and biological environment and unfortunately the literature on nanoparticle dissolution in tissues is sporadic as methodological approaches are recently limited.

The efficient method for the measurement of nanoparticles concentrations in organs is for example inductively coupled plasma mass spectrometry ICP-MS [33], which was used to evaluate in vivo clearance and translocation of europium-doped gadolinium oxide nanoparticles after 24 h nanoparticle instillation [33]. Analysis revealed 59% of the initial nanoparticle dose in the lung, 20% was excreted in the feces, 0.2% was detected in the liver and less than 0.1% of the delivered dose was found in the remaining organs. These results were qualitatively similar to iridium nanoparticles instilled intratracheally in adult rats after 7 days [34], when 59% of the delivered dose was found in the lungs, 35% was excreted in the feces and less than 0.2% found in extrapulmonary organs. These in vivo studies confirmed the presence of intratracheally instilled nanoparticles in liver and the other extrapulmonary organs similar as we observed by transmission electron microscope in all analyzed organs and confirmed also by EDX analysis in case of liver. Therefore, the translocation of nanoparticles through the circulation into secondary organs was confirmed but seems to be low [35], which likely corresponds to our experiments with the majority of nanoparticles observed to stay in the pulmonary tissues. On the other hand, it appears that the accumulation of nanoparticles in target organs might reach a critical threshold causing injury in the case of our sub-chronic exposure as was proposed previously [35].

Based on analysis the ratio of gadolinium to europium ions measured by ICP-MS in several tissue samples [33], we can predict amount of particle and non-particle fraction in different organs. The target Gd:Eu ratio was found to be 4.74 based on the initial precursor concentrations of Gd and Eu nitrates [33]. It means that about 4–5 times higher presence of nanoparticles to ions could be present in our tissue samples, therefore pathological changes observed in organs following inhalation of PbO nanoparticles should be associated preferentially with nanoparticle effect and not dissolved form of lead. Unfortunately, similar study was not performed for lead nanoparticles and as different chemical composition of nanoparticles can affect their behavior in vivo, the ratio of Pb nanoparticles and ions can be altered. Ultimate confirmation of proportional lead content in PbO-NP or non-particulate form will be necessary to obtain by ICP-MS analysis in future.

Blood lead level (BLL, μg/dl) is the biologic index used by health care providers as an indicator of lead exposure. Subclinical lead toxicity remains a problem for both adults and children. In 2012, the Centers for Disease Control and Prevention (CDC) adopted 5 μg/dL as the upper reference level for BLLs in children, representing an advisory level for environmental and educational intervention. In 2015, the National Institute for Occupational Safety and Health (NIOSH) designated 5 μg/dL in whole blood from a venous blood sample as the reference blood lead level for adults. An elevated BLL is defined as a BLL ≥ 5 μg/dL [16]. Blood lead levels of 10 μg/dL are associated with aggressive behaviour and hearing dysfunction in children. On the other hand, there is no detectable threshold for adverse effects of lead on the development of the nervous system [36]. After absorption, lead is distributed to the blood, soft tissues and bones. In the blood, 99% of lead is bound to erythrocytes and 1% exists free in plasma, available for exchange with soft tissues (kidney, brain, liver, bone marrow) [37]. In our study following six weeks of exposure by inhalation to lead oxide nanoparticles, we found 88.2% of the lead in blood cell fraction, a lower proportion (11.6%) in the plasma-protein fraction, and only small proportion in the serum (approximately 0.2%). One of possible explanation of this diversity can be caused by the occurrence of lead in multiple forms where dissolved Pb is probably accumulated in blood cell fraction and PbO nanoparticles interact more with proteins in plasma and thus increasing their level in this blood fraction. The concentration of lead in whole blood of mice exposed for six weeks to PbO-NPs was 132 ng/g (with presumptive density of mouse blood 1 g/ml, blood lead level is 13.2 μg/dL), whereas lead concentration was below the detection limit of 11 ng/g in the control animals. The concentration of lead in blood of our exposed mice exceeded 2.6 times a venous blood sample as the reference blood lead level for adults [16].

Once nanoparticles enter into organs, they penetrate into cells crossing the cytoplasm membrane. Uptake via endocytic routes involves delivery of material into a sub-cellular compartment, the endosome, which is separated from the cell cytoplasm by a membrane. Most endocytic routes end up in degradative compartments of the cell, the lysosomes, where materials are exposed to high concentrations of a wide variety of hydrolytic enzymes. However, there are a few substances, such as heavy metals, which can escape from these recycling activities and accumulate in cells, resulting in toxicity [15]. Our study confirmed such escape of lead oxide nanoparticles from membrane-bounded structures into freely located metal nanoparticles within the cell cytoplasm in all secondary target organs; only in some cases they were located in cytoplasmic vesicles. In the liver, nanoparticles exhibited characteristic arrangement within hepatocyte mitochondria. Previously, the lead has been shown to actively accumulate in the mitochondria [38]. Lead can enter the mitochondria passively at concentrations above 50 μM, inhibiting mitochondrial uptake of Ca^2+^ and causing displacement of Ca^2+^. This effect has been demonstrated in isolated mitochondria or in in situ conditions [38]. Lead oxide nanoparticle accumulation inside hepatocytic mitochondria could function like calcium ions. Similarly, prolonged increase in [Ca2+] leads to the opening of the mitochondrial permeability transition pore (PTP), a critical event leading to cell death by apoptosis [39, 40]. Therefore, lead oxide nanoparticles can cause serious damaging events associated with processes occurring just in cell mitochondria.

Inhalation or intra-tracheal instillation of various nanoparticles was previously shown to cause numerous inflammatory changes in lungs [41–44]. Exposure to nanoparticles resulted in serious lung damage, local or systemic inflammation (assessed by bronchoalveolar lavage), increase in macrophage numbers and influx of neutrophils in bronchoalveolar lavage fluid. Comparison of metal oxide nanoparticles, such as CeO_2_NP, NiONP, ZnONP, and CuONP [11], revealed a unique inflammatory footprint, both acutely and chronically. Thus, nanoparticles cannot be viewed as a single hazard entity and risk assessment should be performed separately for each substance [11].

Moreover, metal oxides of nanoparticles can release their soluble ions in vivo during passages via bronchial tree. These ions were shown to cause diverse effect on individual organs in comparison to their nanoparticle forms [45]. Oxides of zinc in aerosol form attracted specifically neutrophils while ZnO in nanoparticle form attracted preferentially eosinophils. Even further, oxides of nickel in aerosol form caused no inflammatory response in contrast to nanoparticle form inducing serious chronic inflammation. On the other hand, oxides of Cu contributed to the lung inflammation attracting neutrophils into lungs both in aerosol form and nanoparticle form. Unfortunately, similar study comparing the effect of lead oxide nanoparticles and lead oxide salts was not performed yet and this comparison will be necessary to follow in future.

Our sub-chronic study with lead oxide nanoparticles confirmed serious pulmonary inflammation after their inhalation, with the presence of siderophages verifying longstanding pulmonary congestion. Moreover, we observed serious thrombotic events in capillaries and small venules in lung tissue caused by lead oxide nanoparticles inhalation. In secondary target organs, no thromboses were found. As lead can cause imbalance of intracellular calcium in tissues and, in addition, nanoparticles may influence blood coagulation [43, 46], this effect of lead oxide nanoparticles is unsurprising. Whether this coagulation is also connected to increased level of calcium in platelets inducing their aggregation in lungs, however, will be necessary to uncover.

In the liver, we observed excess lipid accumulation following lead exposure, indicating impairment of the normal processes of synthesis and elimination of triglycerides. As the liver is the main organ involved in lipid metabolism, it is commonly associated with steatosis. Therefore, the storage disorders (lipid accumulation) and lysosomal perturbations (reduced membrane stability) represent early markers of toxic effects on liver cells and biomarkers of contaminants such as heavy metals [47]. Inhalation of PbO nanoparticles also caused hydropic degeneration or hypetrophy of hepatocytes, focal necrosis and some areas with inflammation, representing typical features of steatohepatitis [48, 49]. It is thought that hydropic degeneration can be caused by hypoxia, ischaemia or the treatment of hepatocytes with endotoxins or other chemicals [10]. This response has also been observed following exposure to other toxic materials, including copper nanoparticles in mice [50] and cerium oxide nanoparticles in rats [10].

Surprisingly, morphology of lead oxide nanoparticles was altered in the liver where we found clusters of many small-sized (20–30 nm) agglomerates within hepatocyte mitochondria. Based on this observation, we suggest that disintegration or decomposition of lead oxide nanoparticles occurs preferentially in the liver.

High lead concentration did not cause expected serious changes in kidney. In contrast to lung and liver, we have found only modest changes of kidneys in animals exposed to PbO nanoparticles. Areas of inflammatory infiltrates in the renal cortex were observed, predominantly containing lymphocytes or mononuclear cells. However, such cellular infiltrates are routinely observed in the kidneys of rodents and generally increase with age [51]. Thickened filtration membranes with intramembranous electron-dense deposits were observed, similar to kidney tissue exposed to cadmium nanoparticles [52]. Similarly, rats exposed to silver and gold nanometals demonstrated moderate thickening and a clearly enhanced contour of the glomerular basal membranes [53].

Lead oxide nanoparticles were found mostly in epithelial cells of proximal tubules and the presence of nanoparticles was consistent with the highest metabolic activity in the kidney occurring in the proximal tubules. By contrast to hepatocytes in liver or alveolar epithelial cells in lung, lead oxide nanoparticles were sparse in these cells. Interestingly, high concentration of lead as shown by chemical analysis did not corresponded to rare presence of lead oxide nanoparticles in kidney cells. Therefore, we propose that the most of kidney lead was present in its dissolved or metabolized form.

Inhalation of lead oxide nanoparticles caused a significant effect on the histological appearance of the hippocampal region in the mouse brain. We demonstrated significantly increased lead content of the brain after lead oxide nanoparticles exposure. These findings are in accordance with studies by others [30, 54] describing pathological changes mainly in the hippocampal regions of nanoparticle treated animals. Lead intoxication in humans causes axonal degeneration and in some other species it causes a primarily demyelinating neuropathy [55].

Can nanoparticles enter into brain tissues and pass hematoencephalic barrier? After exposure by intranasal instillation, nanoparticles can be deposited in the nasal mucosa and then translocated to the olfactory bulb of the central nervous system (CNS) along the neuronal olfactory route, for example TiO_2_ nanoparticles [56] or copper nanoparticles [12]. Here, lead oxide nanoparticles were able to pass hematoencephalic barrier of brain. In our case, we found lead oxide nanoparticles in neurons and in pericyte cytoplasm, indicating blood-brain barrier translocation. We therefore propose this as the main transport route based on the results of our study, however, we cannot exclude transport via the olfactory route as olfactory nerves were not investigated here.

Lead levels increased approximately 120-fold in the brain cortex following six weeks of PbO nanoparticles exposure. Similarly, exposure to PbS nanoparticles [30] increased lead levels significantly. Lead levels in the cortex were 5.29 times (low dose treatment) and 8.24 times (high dose treatment) higher compared to control samples, and 10.33 times and 13.40 times higher in the hippocampus, respectively. Thus more lead was accumulated in hippocampal area. In this study, male Sprague Dawley rats were exposed to PbS NPs by trachea once every 7 days for 3 consecutive months (15 mg/kg PbS NPs in low exposure group and 30 mg/kg PbS NPs in high exposure group).

It is interesting to note that in our study lead levels in the mouse brain after six weeks of exposure (116 ± 18.13 ng/g) did not reach those in the brain cortex of control animals (0.17 ± 0.04 μg/g) reported by Cao et al. [30]. These discrepancies may be explained by differences between rats and mice, but also by different environmental conditions during both experiments. In spite of this, both studies confirmed increased lead levels in brain cortex after lead nanoparticle exposure.

Lead can cause also imbalances of calcium in nerve tissue [57]. Chronic lead exposure of rats to PbS nanoparticles [30] increased the [Ca^2+^]i in nerve cells in comparison to controls. With increased PbS concentration, memory behaviour of animals was influenced, and average number of errors and escape latency were increased. Our study confirmed the presence of lead oxide nanoparticles in brain, therefore, higher attention should be paid to possible negative effects of these inhaled nanoparticles further. They induce serious changes in brain morphology as they can easily penetrate through brain-barriers.

## Conclusions

In summary, we found significant microscopic as well as ultramicroscopic changes in primary and secondary organs after lead oxide nanoparticle inhalation. In the lungs, nanoparticles formed clusters inside the cytoplasmic vesicles, while in the liver, kidney, spleen and brain they demonstrated tissue-specific subcellular processing. Our study indicates the variability of nanoparticle transport and intake by individual types of cells in different organs as well as unevenness of lead oxide nanoparticle processing by cells, which will be necessary further address in future to reveal and experimentally enhance the mechanism of their clearance.

## Additional file

Additional file 1: Calculation of deposited dose of PbO nanoparticles in the experiments. **Figures S1, S2**: The size distribution of nanoparticles with respect to the number of particles per unit volume in inhaled air and STEM images of PbO nanoparticles collected on TEM grids. **Figures S3, S4**: Weight of organs in the experiments. **Figure S5**: Effect of lead nanoparticles on spleen following 6 weeks exposure to PbO nanoparticles. **Figure S6**: Detection of proliferating cells in brain tissue. **Tables S1, S2**: Lead concentration in organs following 6 weeks exposure in the experiments. **Tables S3, S4**: Pathological changes in kidney, liver and lung in the experiments. (DOCX 4862 kb)
