# Supplementary material for: Sub-chronic inhalation of lead oxide nanoparticles revealed their broad distribution and tissue-specific subcellular localization in target organs
Source: Part Fibre Toxicol. 2017 Dec 21;14:55. doi: 10.1186/s12989-017-0236-y (PMC5740755; doi:10.1186/s12989-017-0236-y)
Supplement: Additional file 1: — Calculation of deposited dose of PbO nanoparticles in the experiments. Figures S1, S2: The size distribution of nanoparticles with respect to the number of particles per unit volume in inhaled air and STEM images of PbO nanoparticles collected on TEM grids. Figures S3, S4: Weight of organs in the experiments. Figure S5: Effect of lead nanoparticles on spleen following 6 weeks exposure to PbO nanoparticles. Figure S6: Detection of proliferating cells in brain tissue. Tables S1, S2: Lead concentration in organs following 6 weeks exposure in the experiments. Tables S3, S4: Pathological changes in kidney, liver and lung in the experiments. (DOCX 4862 kb) [file 12989_2017_236_MOESM1_ESM.docx]

ADDITIONAL FILES

**Sub-chronic inhalation of lead oxide nanoparticles revealed their broad distribution and tissue-specific subcellular localization**

**in target organs**

Dumková J.^1^, Smutná T.^2^, Vrlíková L.^2^, Le Coustumer P.^6,7,8^, Večeřa Z.^3^, Dočekal B.^3^,

Mikuška P.^3^, Čapka L.^3^, Fictum P.^4^, Hampl A.^1^, Buchtová M.^2,5^*

^1^ Department of Histology and Embryology, Faculty of Medicine, Masaryk University, Brno 625 00, Czech Republic

^2^ Institute of Animal Physiology and Genetics, v.v.i., Czech Academy of Sciences, Brno 602 00, Czech Republic

^3^ Institute of Analytical Chemistry, v.v.i., Czech Academy of Sciences, Veveří 97, Brno 602 00, Czech Republic

^4^ Department of Pathological Morphology and Parasitology, Faculty of Veterinary Medicine, University of Veterinary and Pharmaceutical Sciences, Brno 612 42, Czech Republic

^5^ Department of Animal Physiology and Immunology, Institute of Experimental Biology, Faculty of Science, Masaryk University, Brno 625 00, Czech Republic

^6^ Bordeaux University, UF STE, Allée G. Saint-Hilaire, 33615 Pessac Cedex, France

^7^ UMR 5254 IPREM, CNRS/UPPA, Technopole Hélioparc, 2 av P. Angot, 64053 Pau Cedex9, France

^8^ EA 4592 Georessources & Environnement/ Bordeaux Montaigne University-IPNB ENSEGID, Allée F. Daguin, 33615 Pessac Cedex, France

Calculation of deposited dose of PbO nanoparticles in the first experiment

The estimation of deposited dose was calculated based on previously published methodology (Bide et al., 2000; Miller, 2000; Mitchell et al., 2007) and based on the average mass concentration of PbO nanoparticles (121.7 μg PbO/m^3^).

Deposited dose=(C *RMV * T * DF)/BW (Mitchell et al., 2007)

Where C is average concentration in the exposure atmosphere 121.7 µg PbO/m^3^ (121.7ng PbO/L). RMV is respiratory minute volume (L/min), T is exposure time (min) = 60 480 (6 × 7 × 24 × 60). DF is pulmonary deposition fraction (10%), therefore 0.1 (Miller, 2000). BW is average body weight (g) = 24 g. Final calculated deposited dose = 0.749 µg/g PbO.

According to (Bide et al., 2000) respiratory minute volume can be calculated using the equation RMV =0.499 * BW^0.809^ L/min, in which BW holds for body weight (kg) = 0.024 kg.

Estimated dose of PbO was 0.75 μg per gram of mouse body weight over the 6 weeks inhalation period.

Calculation of deposited dose of PbO nanoparticles in the second experiment

The estimation of deposited dose was calculated based on previously published methodology (Bide et al., 2000; Miller, 2000; Mitchell et al., 2007) and based on the average mass concentration of PbO nanoparticles (149.3 μg PbO/m^3^).

Deposited dose=(C *RMV * T * DF)/BW (Mitchell et al., 2007)

Where C is average concentration in the exposure atmosphere 149.3 µg PbO/m^3^ (149.3 ng PbO/L). RMV is respiratory minute volume (L/min), T is exposure time (min) = 60 480 (6 × 7 × 24 × 60). DF is pulmonary deposition fraction(10%), therefore 0.1 (Miller, 2000). BW is average body weight (g) = 24 g. Final calculated deposited dose =0.919 µg/g PbO.

According to (Bide et al., 2000) respiratory minute volume can be calculated using the equation RMV =0.499 * BW^0.809^ L/min, in which BW holds for body weight (kg) = 0.024 kg.

Estimated dose of PbO was 0.92 μg per gram of mouse body weight over the 6 weeks inhalation period.

**REFERENCES**

Bide, R. W., Armour, S. J., & Yee, E. (2000). Allometric respiration/body mass data for animals to be used for estimates of inhalation toxicity to young adult humans. *J Appl Toxicol, 20*(4), 273-290

Miller, F. J. (2000). Dosimetry of particles in laboratory animals and humans in relationship to issues surrounding lung overload and human health risk assessment: a critical review. *Inhal Toxicol, 12*(1-2), 19-57. doi: 10.1080/089583700196329

Mitchell, L. A., Gao, J., Wal, R. V., Gigliotti, A., Burchiel, S. W., & McDonald, J. D. (2007). Pulmonary and systemic immune response to inhaled multiwalled carbon nanotubes. *Toxicol Sci, 100*(1), 203-214. doi: 10.1093/toxsci/kfm196

**Figure S1: The size distribution of nanoparticles with respect to the number of particles per unit volume in inhaled air**

**
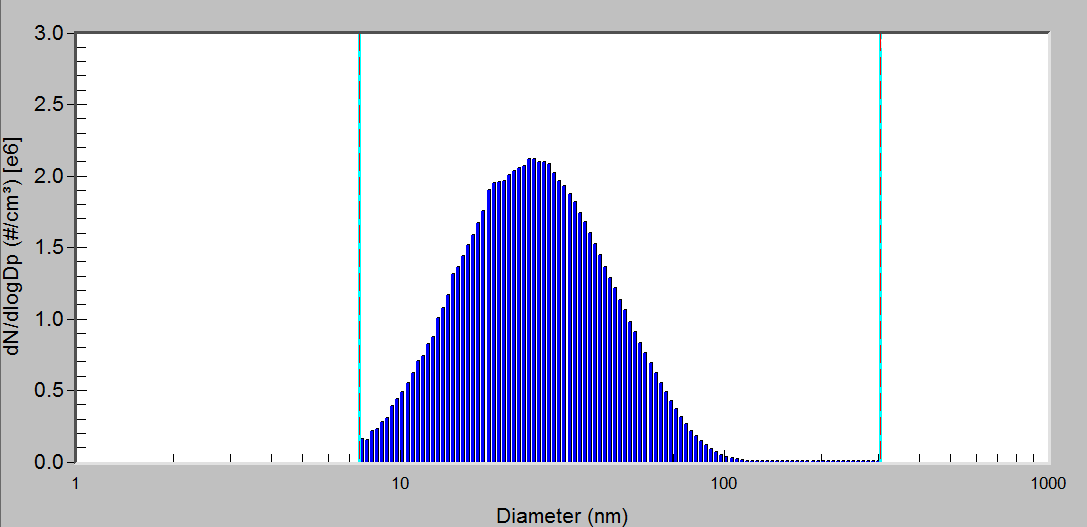
**

**Figure S2: STEM images of PbO-NPs collected on TEM grids**

**
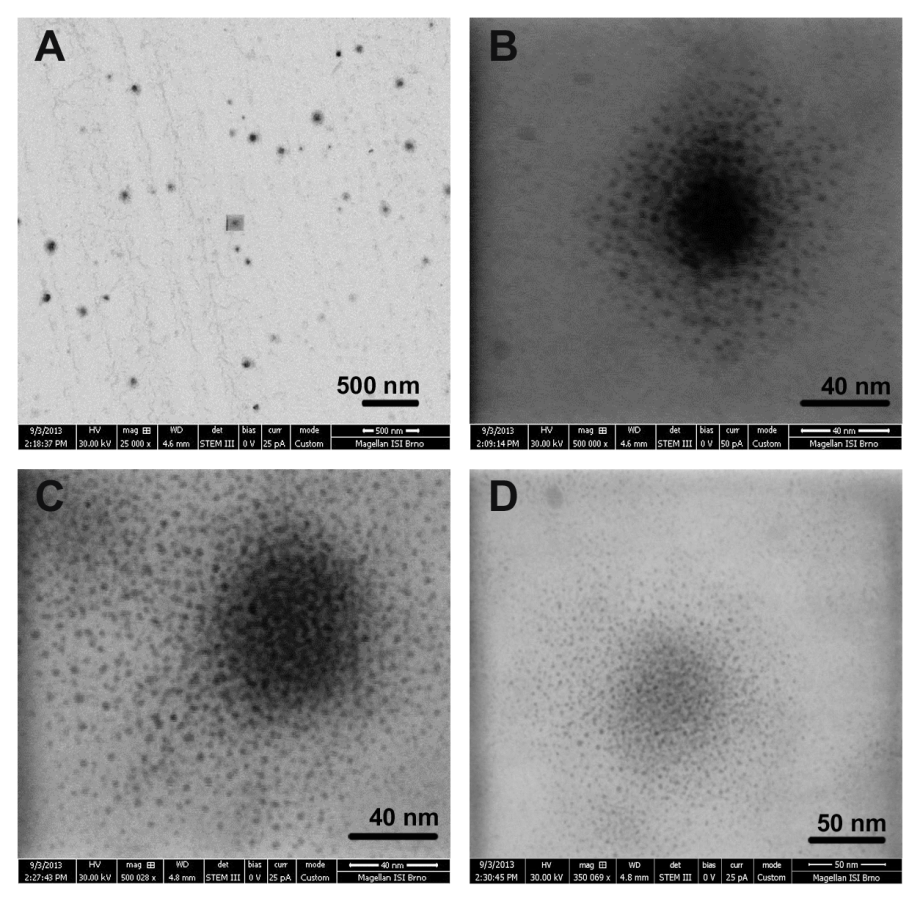
**

**Figure S3: Weight of organs in the first experiment.**

**
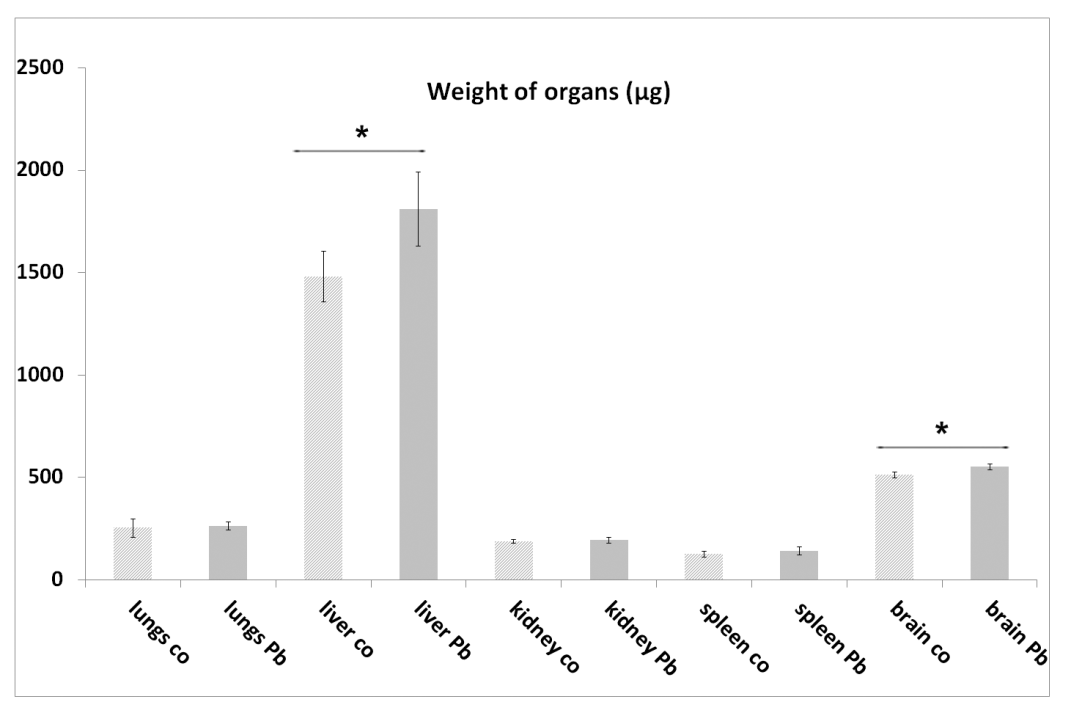
**

The graphs values denote average ± s.d., * p < 0.05 by t-test.

**Figure S4: Weight of organs in the second experiment.**

**
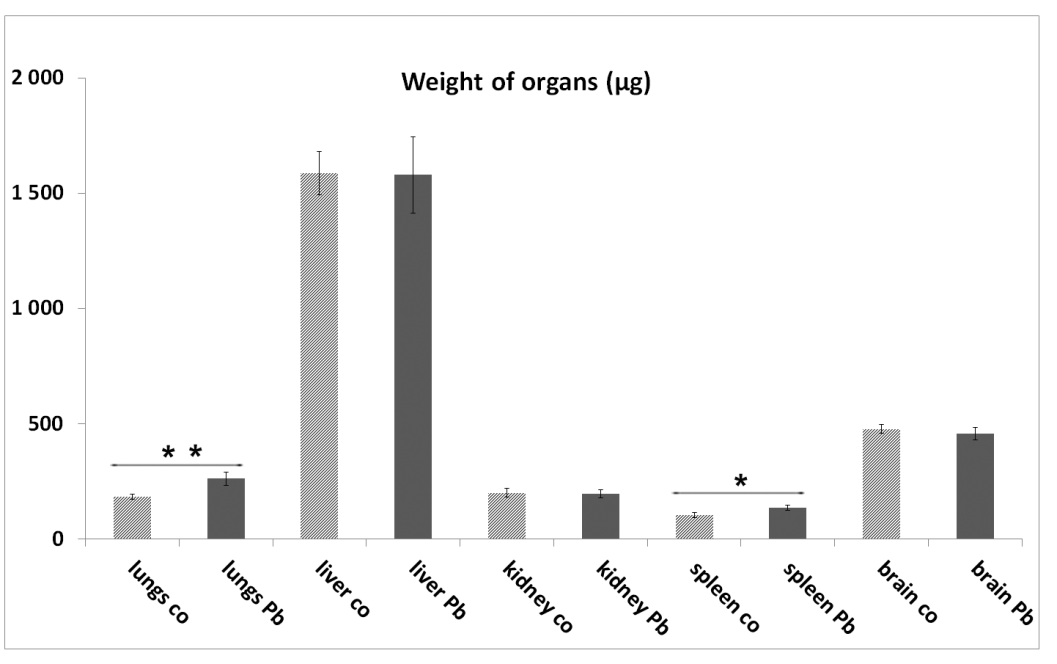
**

The graphs values denote average ± s.d., * p < 0.05 and ** p < 0.01 by t-test.

**Fig. S5 Effect of lead nanoparticles on spleen following 6 weeks exposure** **to PbO nanoparticles**

**A, B)** Control tissues stained with Hematoxylin-Eosin. **C, D)** Lead nanoparticles exposed tissues stained with Hematoxylin-Eosin. Arrows indicate megakaryocytes in splenic pulp. Scale bar in panels A-D = 100 μm. **E-H)** Splenic tissue in transmission electron microscope. **E)** Control sample of splenic pulp with macrophages (ma). **F)** Splenic red pulp with megakaryocyte (me) following lead oxide nanoparticle treatment. **G)** Lead nanoparticles freely located in cell cytoplasm (contrasted section). **H)** Lead nanoparticles freely located in cell cytoplasm (non-contrasted section). Details of lead nanoparticles in the upper corner of panels G, H. Arrowheads show lead oxide nanoparticles.


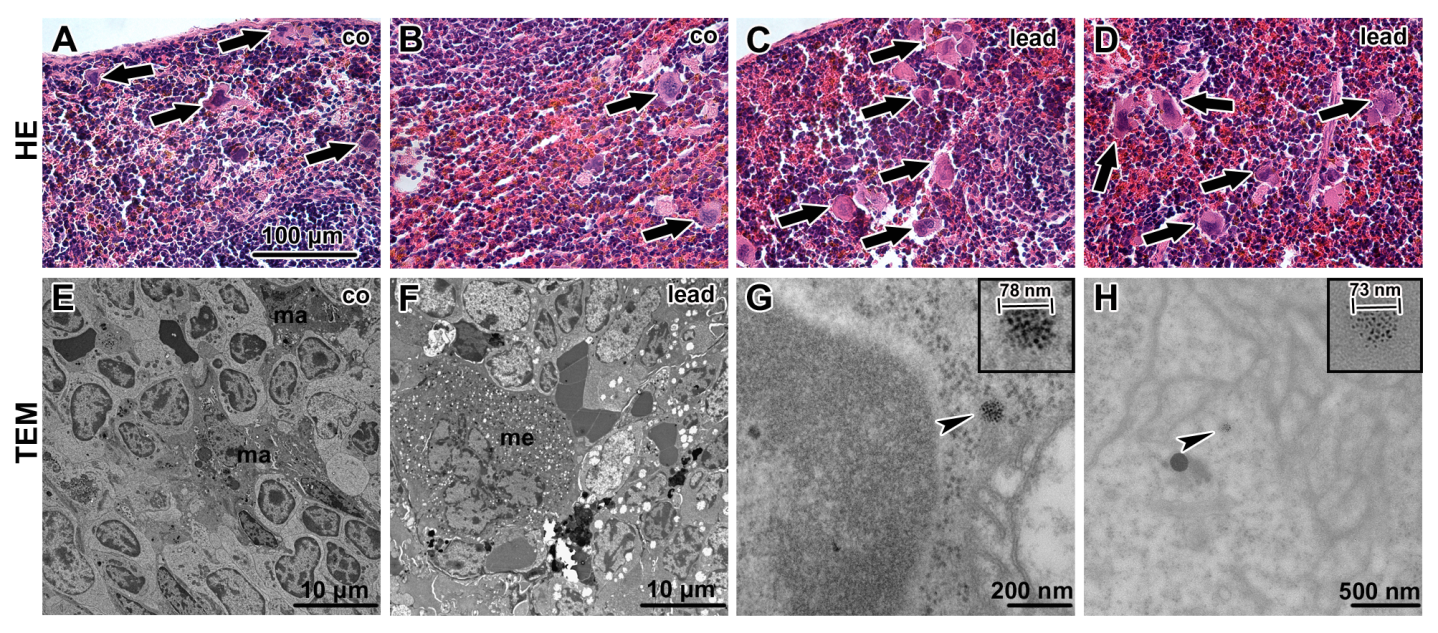


**Figure S6: Detection of proliferating cells in brain tissue**

**A-D)** Brain tissues of control animals with PCNA-labeled cells. **E-H)** Lead oxide nanoparticles exposed brain tissues with PCNA-labeled cells. **A, E)** Hippocampal region of mouse brain. **B, F)** Subgranular zone of dentate gyrus with proliferating neurons. **C, D, G, H)** Subventricular zone with proliferating neurons. Scale bar in panels A, E, C, G = 200 μm. Scale bar in panels B, F, D, H = 100 μm.

**
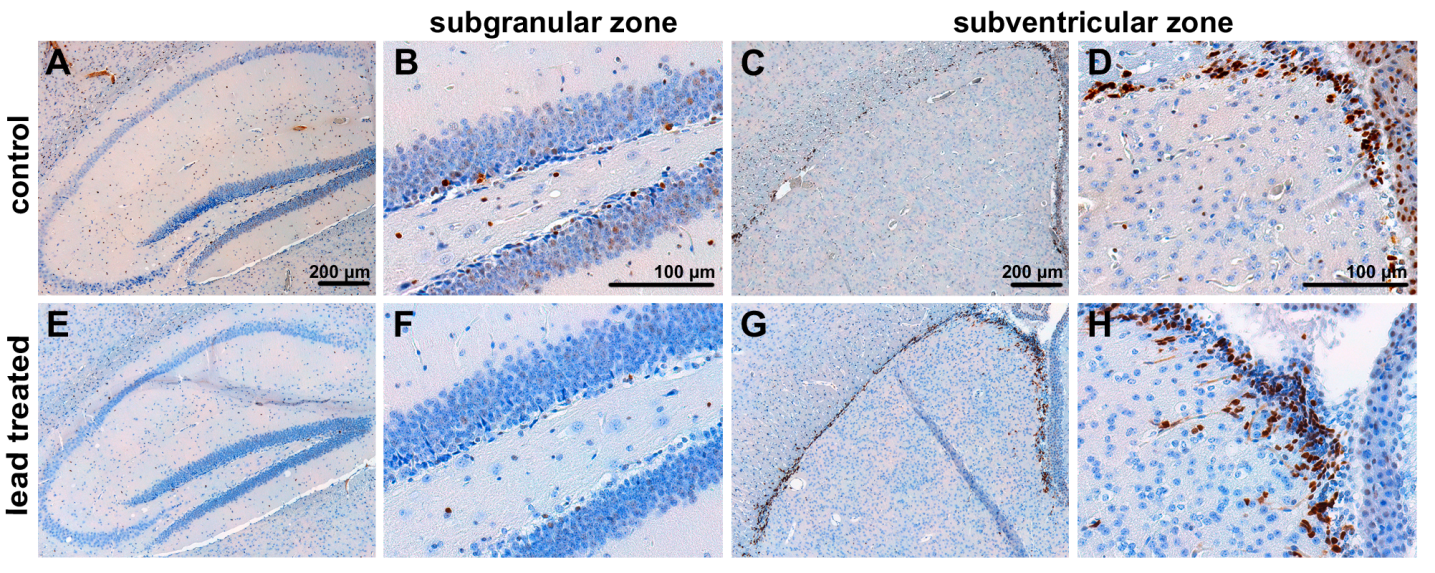
**

**Table S1: Lead concentration in organs following 6 weeks exposure in the first experiment (n=4, mean ± SD)**

| mice | lungs (ng/g) | liver (ng/g) | kidney (ng/g) | spleen (ng/g) | brain (ng/g) |
| --- | --- | --- | --- | --- | --- |
| control | < LOD* | 4±2* | 23±8* | < LOD* | < LOD* |
| exposed | 1485±296 | 543±89 | 3327±614 | 466±115 | 116±18 |

* Limit of detection in lung, liver, kidney, spleen and brain tissue was 19, 3, 14, 17 and 8 ng/g Pb, respectively.

**Table S2: Lead concentration in organs following 6 weeks exposure in the second experiment (n=3, mean ± SD)**

| mice | lungs (ng/g) | liver (ng/g) | kidney (ng/g) | spleen (ng/g) | brain (ng/g) |
| --- | --- | --- | --- | --- | --- |
| control | < LOD* | < LOD* | < LOD* | < LOD* | < LOD* |
| exposed | 2147±50 | 541±27 | 2585±240 | 519±62 | 109±20 |

* Limit of detection in lung, liver, kidney, spleen and brain tissue was 33, 3, 21, 67 and 41 ng/g Pb, respectively.

**Table S3: Pathological changes in kidney, liver and lung in the first experiment**

| kidney | K1 | K2 | K3 | K4 | K5 | Pb1 | Pb2 | Pb3 | Pb4 | Pb5 |
| --- | --- | --- | --- | --- | --- | --- | --- | --- | --- | --- |
| infl. cell inf. perivasc. |  |  |  |  |  | ++ | ++ | + | ++ |  |
| infl. cell inf. peritub. |  |  |  |  |  | ++ | ++ | + | ++ |  |
| infl. cell inf. in pelvis |  |  |  |  |  |  |  |  |  |  |
| enlarged JM glomeruli | + | + | + | + | + | + | + |  | + | + |
| glom. metaplasia |  |  |  |  | + | + |  |  | + |  |
| infl.cell inf. in medulla |  |  |  |  |  | + | + |  | + |  |
| liver | K1 | K2 | K3 | K4 | K5 | Pb1 | Pb2 | Pb3 | Pb4 | Pb5 |
| mononucl. cell inf. | ++ | + | + | + | + | + | + | + | + | + |
| focal necrosis |  |  |  |  |  |  |  |  |  | + |
| vacuolated hep. |  |  |  |  |  | + |  |  | + |  |
| increase of binucl.hep. |  |  |  |  |  |  |  |  | + |  |
| aff. sinus., hemostase |  |  |  |  |  | + | + |  | + |  |
| hep. remodeling |  |  |  |  |  | + | + |  | + |  |
| hypertrophic hep. |  |  |  |  |  |  | + |  | ++ |  |
| lung | K1 | K2 | K3 | K4 | K5 | Pb1 | Pb2 | Pb3 | Pb4 | Pb5 |
| infl. cell inf. peribron. | + |  |  |  |  | + |  |  |  |  |
| atelectasis |  |  |  |  |  |  |  |  | + |  |
| bronchiolitis |  |  |  |  |  |  | + |  | + |  |
| hyperemia, cong. cap. |  |  |  |  |  |  |  | + | + |  |
| alveolar emphysema | + | + |  |  |  |  | ++ |  | + | ++ |
| bronchiectasia |  |  |  |  |  |  |  |  |  | ++ |

We evaluated at least 8-10 slides per organ and assessed alterations in patho-histological changes as follows: **kidney** – inflammatory cell infiltrate perivascular, inflammatory cell infiltrate peritubular, inflammatory infiltrate in pelvis, enlarged juxtamedullary glomeruli, glomerular metaplasia, higher cellularity in glomeruli, inflammatory infiltrate in medulla; **liver** – mononuclear cell infiltrate, focal necrosis (degenerating hepatocytes), vacuolated hepatocytes, increase of binucleated hepatocyte, affected sinusoids with hemostase, hepatic remodeling, centrilobular hypertrophic hepatocytes; **lung** – inflammatory cell infiltrate peribronchiolar, inflammatory cell infiltrate perivascular, atelectasis, bronchiolitis, hyperemia with congested capillaries, alveolar emphysema, hemostase with presence of siderophages, bronchiectasia.

Increased level of phenotype is labelled by increased number of + symbols, where "+" means mild phenotype and "++" moderate phenotype in relevant type of alteration in organ;

K1-K5 control animals, Pb1-Pb5 lead exposed animals

**Table S4: Pathological changes in kidney, liver, lung and brain in the second experiment**

| kidney | K6 | K7 | K8 | K9 | K10 | Pb6 | Pb7 | Pb8 | Pb9 | Pb10 |
| --- | --- | --- | --- | --- | --- | --- | --- | --- | --- | --- |
| infl. cell inf. perivasc. |  |  |  |  | + |  |  |  |  |  |
| infl. cell inf. peritub. | ++ |  |  | + | ++ | + | + | + | + | + |
| infl. cell inf. in pelvis | ++ |  |  | ++ | + | + | ++ | + |  | + |
| enlarged JM glomeruli |  | + |  | + |  |  | + |  |  |  |
| glom. metaplasia |  |  |  |  |  |  |  |  | + | + |
| higher cellul. in glom. |  |  |  |  |  |  |  | ++ | + | + |
| infl.cell inf. in medulla |  |  |  |  |  |  |  | + |  |  |
| liver | K6 | K7 | K8 | K9 | K10 | Pb6 | Pb7 | Pb8 | Pb9 | Pb10 |
| mononucl. cell inf. |  |  | + | + |  | + | + | + | + |  |
| focal necrosis |  |  |  |  |  | + | + | + | + | + |
| vacuolated hep. |  |  |  |  |  | + | + |  |  | + |
| increase of binucl.hep. |  |  |  |  |  |  | + | + | + | + |
| aff sinus., hemostase |  |  |  |  |  |  | + |  |  | + |
| hep. remodeling | + |  | + |  | + |  | + | + |  | + |
| hypertrophic hep. |  |  |  |  |  |  | + |  |  |  |
| lung | K6 | K7 | K8 | K9 | K10 | Pb6 | Pb7 | Pb8 | Pb9 | Pb10 |
| infl. cell inf. peribron. | + |  |  | + | + | + | + | + | + | + |
| infl. cell inf. perivasc. |  |  |  |  |  | + | + | + | ++ | + |
| atelectasis |  |  |  |  |  |  | + |  | + |  |
| bronchiolitis | + |  |  | + |  | + |  |  | + |  |
| hyperemia, cong. cap. | + |  |  | + |  | ++ | + | + | + | + |
| alveolar emphysema |  |  |  |  |  |  | + |  | + |  |
| hemostase with sider. |  |  |  |  |  | + |  | + | + |  |
| brain | K6 | K7 | K8 | K9 | K10 | Pb6 | Pb7 | Pb8 | Pb9 | Pb10 |
| necrotic nn in hippoc. | + |  |  |  |  |  | ++ | + | ++ | + |
| spongiform changes | + |  |  |  | + |  | ++ | + | ++ | + |
| vacuoles | + | + | + | + |  |  | + | + | + | + |

We evaluated at least 8-10 slides per organ and assessed alterations in patho-histological changes as follows: **kidney** – inflammatory cell infiltrate perivascular, inflammatory cell infiltrate peritubular, inflammatory infiltrate in pelvis, enlarged juxtamedullary glomeruli, glomerular metaplasia, higher cellularity in glomeruli, inflammatory infiltrate in medulla; **liver** – mononuclear cell infiltrate, focal necrosis (degenerating hepatocytes), vacuolated hepatocytes, increase of binucleated hepatocyte, affected sinusoids with hemostasis, hepatic remodeling, centrilobular hypertrophic hepatocytes; **lung** – inflammatory cell infiltrate peribronchiolar, inflammatory cell infiltrate perivascular, atelectasis, bronchiolitis, hyperemia with congested capillaries, alveolar emphysema, hemostasis with presence of siderophages, bronchiectasis; **brain** - necrotic neurons in hippocampus, spongiform changes, presence of vacuoles in neurons.

Increased level of phenotype is labelled by increased number of + symbols, where "+" means mild phenotype and "++" moderate phenotype in relevant type of alteration in organ;

K6-K10 control animals, Pb6-Pb10 lead exposed animals – in the second independent experiment
